# Desert‐like badlands and surrounding (semi‐)dry grasslands of Central Germany promote small‐scale phenotypic and genetic differentiation in *Thymus praecox*

**DOI:** 10.1002/ece3.5844

**Published:** 2019-12-02

**Authors:** Kevin Karbstein, Salvatore Tomasello, Kathleen Prinz

**Affiliations:** ^1^ Institute of Ecology and Evolution of Plants Systematic Botany with Herbarium Haussknecht and Botanical Garden Friedrich‐Schiller‐University Jena Jena Germany; ^2^ Department of Systematics, Biodiversity and Evolution of Plants (with Herbarium) Albrecht‐von‐Haller Institute for Plant Sciences University of Goettingen Goettingen Germany; ^3^ Landschaftspflegeverband Suedharz/Kyffhaeuser e.V. Nordhausen Germany

**Keywords:** badlands, Central Germany, genetic differentiation, grasslands, phenotypic differentiation, *Thymus praecox*

## Abstract

Environmental heterogeneity among sites can generate phenotypic and genetic variation facilitating differentiation and microevolution of plant populations. Badlands are desert‐like, predominantly vegetation‐poor habitats often embedded in (semi‐)dry grasslands. The desert‐like conditions of badlands demand extreme adaptation of plants, that is, phenotypic modifications in short‐term and/or natural adaptation in long‐term. However, detailed knowledge is missing about both plant phenotypic and genetic differentiation in this unique and widely occurring habitat type. The present study focused on the largest known badlands systems in Central Europe located in the “Drei Gleichen” region, a designated nature conservation area in Central Germany. Locations were suitable for this study in terms of having co‐occurring badlands and (semi‐)dry grassland habitats (sites) occupied by the pioneer plant *Thymus praecox*. Here, we studied the environmental preferences, morphological and functional trait variation, and genetic variation using microsatellite markers of *T. praecox*. Results revealed significant, mainly site‐dependent environmental, phenotypic, and genetic differentiation. In general, individuals in badlands are shorter in height and have lower patch sizes (length × width), relative growth rates, and smaller stomata. The PCA additionally unveiled slightly increased leaf robustness, trichome density, decreased stomatal conductance, fewer females, and earlier phenology in badlands. We interpret differentiation patterns as adaptive responses to light, temperature, drought, and nutrient stress conditions supported by reviewed literature. Genetic differentiation was strongest between local badlands and grassland sites, and clearly weaker among locations and between sites (in total) as indicated by *G*_ST_, AMOVA, PCoA, and population structure. Our study supports the importance of small‐scale microhabitat conditions as a driver of microevolutionary processes, and the population's need for sufficient phenotypic variation and genetic resources to deal with environmental changes. We demonstrated that badlands are an appropriate model system for testing plant response to extreme habitats and that more research is needed on these fascinating landscapes.

## Graphical abstract

Badlands, that is, desert‐like and widely vegetation‐poor habitats, demand extreme adaptation of plants. Focussing on the largest known badlands systems in Central Europe, we investigated small‐scale phenotypic and genetic differentiation in *Thymus praecox* between local badlands and surrounding grassland sites.

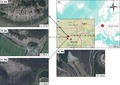

## 1 INTRODUCTION

Badlands, open and desert‐like habitats at steep slopes often embedded in grassland landscapes, represent an example of very heterogeneous environments. Wind and water erosion are responsible for almost no organic soil layer leading to sparse vegetation cover (Guàrdia, Gallart, & Ninot, 2000; Regües et al., 2017; Regüés, Guàrdia, & Gallart, 2000; Torri, Calzolari, & Rodolfi, 2000). Badlands are usually found in arid, semi‐arid to Mediterranean regions, for example in the Badlands National Park (USA), Crete Senesi (Italy), and Las Médulas (Spain). Plants inhabiting these landscapes have to cope with extreme conditions such as high solar radiation, temperature fluctuations, and drought. The desert‐like conditions of badlands demand extreme adaptation of plants, that is, phenotypic modifications and/or adaptation through natural selection. However, despite the worldwide occurrence, and in contrast to vegetation (composition) studies, both phenotypic and genetic differentiation of plants growing in badlands have not been investigated in detail so far (Guàrdia et al., 2000; Regüés et al., 2000, 2017).

Some of the most prominent Central European badlands are located in the “Drei Gleichen” (castle) region between Gotha and Arnstadt (Thuringia, Central Germany). These badlands are embedded in semi‐natural grassland landscapes and have been formed through anthropogenic land use by sheep‐ and goat‐grazing presumably since medieval ages (Klug, 2006, 2010, 2014; Kräham, 2002; Werneburg, 2003; see Figures S1–S3). The sparse vegetation is composed of regional pioneer and ruderal species together with elements of subcontinental and submediterranean regions and continental steppes (e.g., *Oxytropis pilosa*, *Stipa capillata*, and *Adonis vernalis*; Kräham, 2002; Klug, 2006, 2010; Culmsee, Herrling, Schmiedel, Schwienheer, & Wolf, 2010).

Badlands habitats are rare in Central Europe. Particular geological, geomorphological, climatic, and anthropogenic factors are necessary to produce and preserve badlands formations. The soil types in the Drei Gleichen region originated in the Upper Triassic epoch and consist mostly of red‐brown siliceous clay stones and gray‐green to dark gray calcareous marlstones susceptible to erosion (Klug, 2010; Werneburg, 2003). On steep, mostly southern slopes, strong rainfalls easily weather the bedrock leading to erosion dunes and gullies. Thus, soil development is almost impossible. The absence of organic soil layer leads to low water retention capacity, extended dry periods, and high temperatures in summer (>60°C, few centimeters above the soil, personal observation) as well as strong soil frost in winter (Klug, 2010; Kräham, 2002). Due to tectonic reasons and erodibility, soils consisting of Upper Triassic stones on steep, southern slopes are rarely found in Central Europe (see Meschede, 2015). Furthermore, Central Germany (“Thüringer Becken”) is one of the driest regions in Germany with less than 540 mm annual precipitation (https://en.climate-data.org, accessed 29 May 2019, 1982–2012) and thus more prone to badlands formations.

Phenotypic plasticity is thought to be one of the major means by which a plant can deal with environmental variability and is defined as the phenotypic expressions of a single genotype under different environmental conditions (Gratani, 2014; Hufford & Mazer, 2003; Sultan, 2000). Phenotypic plasticity is believed to have evolved as a mechanism for adaptation to variable environments maximizing individual's fitness, for example, in enabling colonization of novel habitats by different niche exploitation (Agrawal, 2001; Chevin & Hoffmann, 2017; Dudley & Schmitt, 1996; Via et al., 1995; Violle et al., 2012). Phenotypic plasticity plays different roles in relation to the plant's response to environmental differences, that is, morphological (together with anatomical) and physiological plasticity (Arnold, Kruuk, & Nicotra, 2019; Gratani, 2014). Morphological and anatomical plasticity influences resource acquisition and plant competitive abilities: Taller plants are able to put their leaves over smaller ones, pre‐empt light resources, and outcompete them (Cornelissen et al., 2003; Díaz et al., 2016; Moles et al., 2009). In spatially and temporally variable environments, physiological variability may allow individuals to grow and to reproduce better (Ackerly et al., 2000; Griffin, Epstein, & Boelman, 2013). For example, stomatal development, among other factors, varies with CO_2_ concentration, light intensity, and water supply (Casson & Gray, 2008; Lau & Bergmann, 2012; Woodward, Lake, & Quick, 2002), enabling individuals to regulate their metabolism and to optimize their stomatal conductance.

Despite different roles for plant response, several studies indicate a correlation between phenotypic plasticity/variation and genetic variation for morphological (Waitt & Levin, 1998) and physiological traits (Ackerly et al., 2000; Buckler, Gaut, & McMullen, 2006). Evolution of phenotypic plasticity is possible (depending on the availability of required genetic variation) if a genetic correlation with traits under selection or genetic drift exists (Agrawal, 2001; Van Kleunen & Fischer, 2007; Via et al., 1995; Via & Lande, 1987). However, genetic variation needs not necessarily to coincide with plasticity (Chevin & Hoffmann, 2017). Moreover, functional traits, which are morphological and ecophysiological traits affecting an individual's fitness indirectly via growth, reproduction, and survival (Nock, Vogt, & Beisner, 2016; Violle et al., 2007), have been also shown to vary with not only environmental conditions but also with genetic variation within species (Bernhardt‐Römermann et al., 2011; Bucher et al., 2016; Karbstein, Prinz, Hellwig, & Römermann, submitted.; Violle et al., 2012). Therefore, functional traits are appropriate to study population responses to environmental variability (see also Sakaguchi et al., 2018).

In an extremely variable environment, a population could become both more plastic and more genetically variable (Gratani, 2014). Dozens of studies done over the previous decades indicate that environmental variability (e.g., in temperature, light intensity, soil characteristics, land use, pollinators, and parasitism) can promote phenotypic and genetic variation in plant populations, as well as differentiation among  them (Linhardt & Grant, 1996; Sakaguchi et al., 2018; Sultan, 2000; Turesson, 1922). For example, genetically uniform *Polygonum lapathifolium* individuals grown in dry and flooded soils exhibited remarkably contrasting phenotypes (Sultan, 2000), and genetically diverse *Trifolium montanum* populations showed high intraspecific functional trait variation within highly variable semi‐dry calcareous grassland habitats (Karbstein et al., submitted).

Even within geographically small scales, environmental heterogeneity promotes plant phenotypic and genetic differentiation. Genetic variation with corresponding phenotypic (ecotypic) differentiation caused by habitat differences was found, for example, between ~100 km distant *Solidago virgaurea* serpentine and nonserpentine populations in Japan (Sakaguchi et al., 2018), between one to 17 km distant *Ranunculus acris* semi‐natural and agriculturally improved grassland populations in Central Germany (Odat, Jetschke, & Hellwig, 2004), or even between populations separated by less than few meters and/or centimeters that were under high selective pressure (Linhardt & Grant, 1996). Additionally, heterogeneous conditions at small scale also influence genetic differentiation by changing gene flow. Geographical barriers, pollinator limitation, and genetic drift can alter gene flow, population connectivity, and thus genetic diversity (Freeland, Kirk, & Petersen, 2011; Heywood, 1991; Linhardt & Grant, 1996).

On the one hand, phenotypic modifications allow for (reversible) short‐term responses increasing individual's fitness in changing environments (e.g., climate or land use). On the other hand, genetic variation promotes long‐term adaptation and evolutionary persistence. When selective pressure acts consistently on specific traits favorable in the specific environment, environmental heterogeneity can lead to the formation of locally differentiated, environmentally adapted genotypes (ecotypes; Gratani, 2014; Hufford & Mazer, 2003; Sakaguchi et al., 2018).

*Thymus praecox* Opiz (Lamiaceae) is a pioneer plant species in badlands and surrounding (semi‐)dry grasslands. The economically important genus *Thymus* is known for its genetic compatibility, and thus sympatrically occurring species can cross with each other (Sostaric et al., 2012). Population variability of tetraploid species in the section *Serpyllum* (e.g., *T. praecox*) is high in terms of morphological traits and chemical composition in secondary metabolites (Ali, Guetat, & Boussaid, 2012; Bączek, Pióro‐Jabrucka, Kosakowska, & Węglarz, 2019; Dajic Stevanovic, Sostaric, Marin, Stojanovic, & Ristic, 2008; Lisi, Tedone, Montesano, Sarli, & Negro, 2011; Rota, Herrera, Martínez, Sotomayor, & Jordán, 2008). Reticulate evolution, that is, hybridization (allopolyploidization) between several diploid progenitor species, presumably caused the observed high variability (Jalas & Kaleva, 1970; Sostaric et al., 2012; Stahl‐Biskup & Sáez, 2002). To the best of our knowledge, only a few studies focused on population genetics of species within the genus *Thymus*. Population diversity, genetic relationships, and local adaptation have been studied using genetic markers (allozyme, AFLP, and RAPD; Ali et al., 2012; Echeverrigaray et al., 2001; López‐Pujol, Bosch, Simon, & Blanché, 2004; Sostaric et al., 2012). Moreover, *T. praecox* is also morphologically and genetically highly variable (Landergott, Naciri, Schneller, & Holderegger, 2006; Schmidt, 1968). Six subspecies have been described (Jalas, 1972; Stahl‐Biskup & Sáez, 2002) for *T. praecox*, with *T. praecox* subsp. *praecox* occurring in Central German badlands. Although several chemotypes are known for different subspecies of *T. praecox* (Bischof‐Deichnik, Holtuijzen, & Stahl‐Biskup, 2000; Stahl‐Biskup, 1986), there is a lack of knowledge on both phenotypic and genetic differentiation.

Considering the noticeable environmental differences between badlands and surrounding (semi‐)dry grasslands, we hypothesize that microhabitat differences led to the formation of site‐specific phenotypes with corresponding genetic differentiation in *T. praecox*. In this study, we therefore aim at understanding the occurrence and process of phenotypic and genetic responses to remarkable different site‐specific microhabitat conditions. We tested our hypothesis by assessing environmental factors, phenotypic (morphological and functional) traits, and genetic diversity using microsatellite markers, at sites of different study locations.

## 2 MATERIAL AND METHODS

### 2.1 Study species

*Thymus praecox* Opiz (Lamiaceae) is a perennial dwarf shrub growing up to 15 cm (Schmidt, 2011). The species is characterized by a procumbent, basal lignified monopodium with inflorescences ending in lateral and long creeping sterile shoots (Jalas & Kaleva, 1970; Schmidt, 1968, 2011). The pedicel is weakly tetragonal, rather round and completely covered with trichomes. Trichome density of ovate to spatulate and leathery leaves is highly variable (Schmidt, 1968, 2011; Schmidt, pers. com.). *Thymus praecox* flowers from May to July in terminal head‐shaped inflorescences consisting of five to 40 zygomorphic, gynodioecious, and self‐compatible flowers (Owens & Ubera‐Jiménez, 1992; Schmidt, 2011). Sex determination, studied on the closely related *Thymus vulgaris*, is complex and regulated by an interplay of nuclear, cytoplasmatic, and environmental factors (Dommée & Jacquard, 1985; Manicacci, Couvet, Belhassen, Gouyon, & Atlan, 1996). Female flowers of *T. vulgaris* are in general smaller than hermaphroditic ones, but the size variation of female flowers depends on the degree of stamen abortion (Thompson, Rolland, & Prugnolle, 2002). Insects such as bees, bumblebees, hoverflies, and beetles can pollinate flowers of *T. praecox*. We observed that seeds are dispersed via endozoochory (sheep, goats; Engler et al., 2009) and anemochory (particular in wind‐exposed badlands sites), but they can be also myrmecochorously (Schmidt, 2011) dispersed over short distances. The species is tetraploid with 2*n* = 50–58 (Jalas, 1972; Jalas & Kaleva, 1967; Pigott, 1955; Rice et al., 2015; Schmidt, 1968). A variable chromosome number might be the result of a reticulate evolution of *T. praecox* (Sostaric et al., 2012). The species mainly grows in calcareous (semi‐)dry *Festuco‐Brometea* grasslands, as well as on calcareous colline to alpine rock and gravel areas in Central Europe (Meusel & Jäger, 1998; Schmidt, 2011). The occurrence of *T. praecox *is fragmented in Germany, with the main distribution in Central and Southern Germany (GBIF Secretariat, 2017; Meusel & Jäger, 1998).

### 2.2 Study region and population sampling

**Figure 1:**
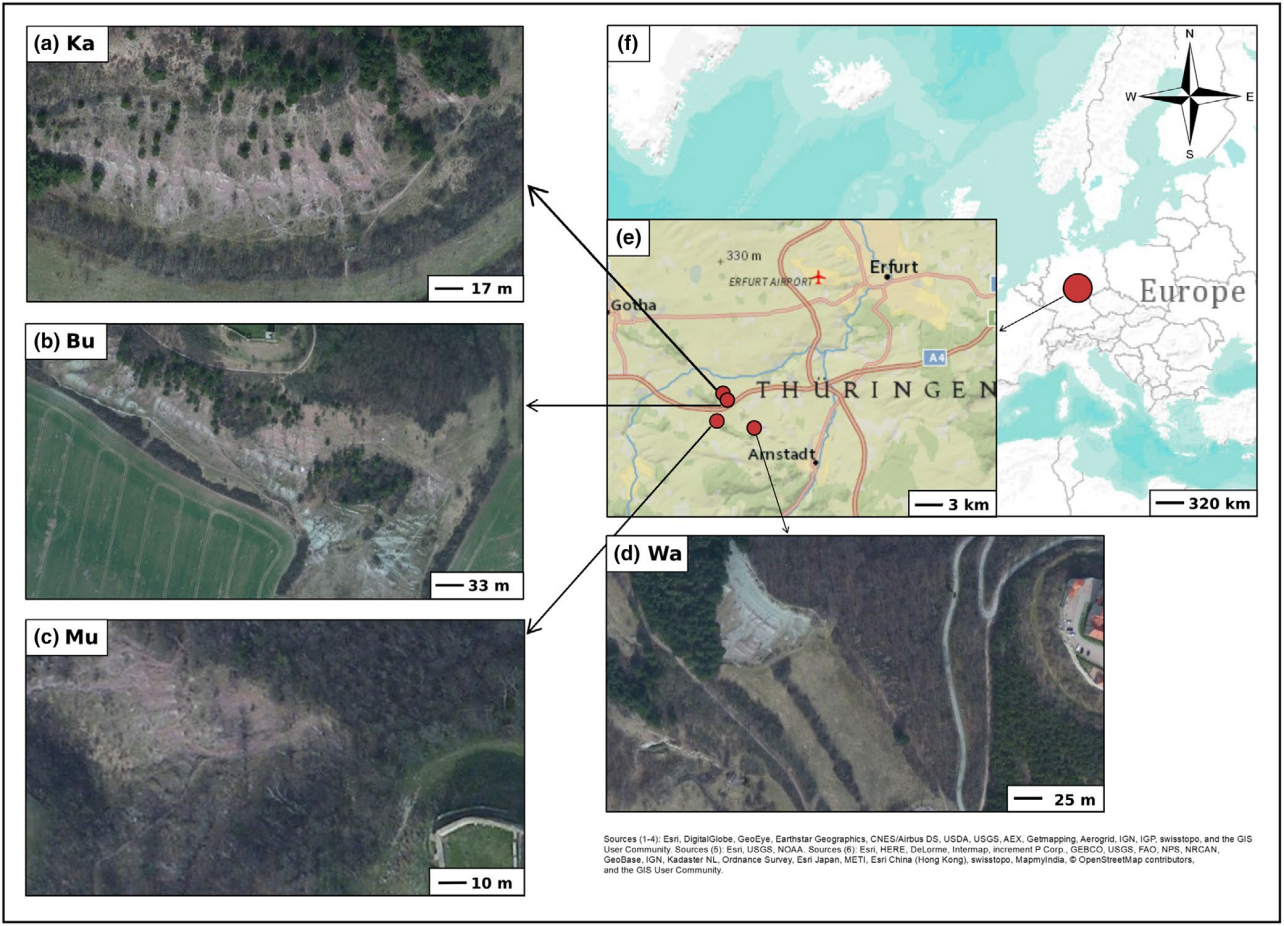
Study area including locations Kallenberg (a, Ka), Burg Gleichen (b, Bu), Mühlburg (c, Mu), and Wachsenburg (d, Wa) in the Drei Gleichen region of Thuringia (e), Central Germany (f). Each location consists of badlands (B) and grassland (G) sites. The basic map was created with ArcGIS vers. 10 (ESRI, Redlands, USA) Sources: 1‐4: (a)‐(d), 5: (e), and 6: (f).

We focused our study on four locations in the Drei Gleichen region between Gotha and Arnstadt, which has been a designated protected area for several decades (Thuringia, Central Germany, Figure 1): Kallenberg (Ka; 50.885069N, 10.84201E) and Burg Gleichen (Bu; 50.879174N, 10.839317E) near Wandersleben, Mühlburg near Mühlberg (Mu; 50.869736N, 10.830219E), and Wachsenburg near Holzhausen (Wa; 50.857818N, 10.873177E). These four locations were suitable for the present study because each location has neighboring badlands (B) and grassland (G) sites occupied by *T. praecox* subsp. *praecox* (Figure 1, see Figures S1–S3, Table S8 for pairwise geographical distances). The Drei Gleichen region is characterized by an annual mean temperature of 8.0°C, approximately 1,500 hr of sunshine and an annual mean precipitation of 540 mm, rather typical of a continental climate (sunshine: https://www.dwd.de, accessed 29 May 2019, 1961–1990; precipitation and temperature: https://en.climate-data.org, accessed 29 May 2019, 1982–2012).

We used the term “location” for main study areas named after the closest villages or hills. The term “site” was applied to separate areas with different biotic and abiotic conditions (Bresinsky, Körner, Kadereit, Neuhaus, & Sonnewald, 2008). Prior to sampling and field measurements, we estimated vegetation cover in each location (estimated area: Ka: 10,500 m^2^, Bu: 41,000 m^2^, Mu: 4,900 m^2^, Wa: 19,000 m^2^) and categorized each location into (a) badlands with a vegetation cover equal or less than 15% and (b) grasslands with a vegetation cover equal or higher than 35% (see also Figure 1). Hence, we ignored individuals in transition zones to avoid ambiguous sampling. In most locations, there was only one vast badlands site surrounded by grasslands (except at Bu). Badlands and grassland sites always occurred adjacent to each other (see Figure 1). There are no physical barriers between sites. Distances were always <100 m between central points of local sites. Therefore, distances were relatively low between sampled *T. praecox* individuals of badlands and grasslands (ca. 1–100 m). If available, we sampled 20 individuals per site. We only collected individuals that were clearly separated from each other reducing the risk of sampling clonal ramets. The sample size of individuals at badlands in Mühlburg (Mu) and Wachsenburg (Wa) is lower because only a few individuals of *T. praecox* occupied these sites (see Table 1).

**Table 1:** Genetic diversity and differentiation indices of populations at locations Kallenberg (Ka), Burg Gleichen (Bu), Mühlburg (Mu), and Wachsenburg (Wa), at badlands and grassland (B and G) sites and at local sites (Location + Site) based on 109 *Thymus praecox* individuals

	Group	*N*	*N*_A_	*P*_Ap_ [%]	*H*_e_	*uh*	*G*_ST_
Location	Ka	39	87	72.4	0.839	0.179	0.015
	Bu	30	84	71.6	0.832	0.172	
	Mu	19	69	59.5	0.852	0.180	
	Wa	21	86	74.1	0.870	0.184	
Site	B	47	95	81.9	0.857	0.180	0.003
	G	62	104	89.7	0.861	0.185	
Location + Site	KaB	19	70	80.5	0.828	0.173	0.035
	KaG	20	69	79.3	0.822	0.169	
	BuB	15	69	82.1	0.829	0.178	
	BuG	15	67	78.6	0.804	0.161	
	MuB	7	53	75.4	0.824	0.184	
	MuG	12	58	84.1	0.836	0.164	
	WaB	6	38	41.9	0.802	0.141	
	WaG	15	77	89.5	0.861	0.188	

Abbreviations: *G*_ST_, genetic differentiation of subpopulations relative to the whole populations; *H*_e_, expected heterozygosity; *N*_A_, allelic richness; *P*_Ap_, percent of private alleles; *uh*, Nei's unbiased genetic diversity.

We collected two to three shoots and wrapped them in wet paper towels. We stored shoots at 7–8°C for phenotypic trait analyses and additionally dried two to three shoots in silica gel for population genetic analyses. In total, we sampled 125 *T. praecox* individuals at four locations from June to July 2014.

### 2.3 Environmental analyses

In each site, we recorded abiotic and biotic environmental factors based on three vegetation records (2 m × 2 m) at a maximum, yielding to six vegetation records in each location and 25 records in total. We calculated species richness, used the extended Braun–Blanquet scale (*r*, +, 1, 2m, 2a, 2b, 3, 4, 5; Maarel, 1979) to estimate species abundance, and recorded cover of herb layer [%], GPS‐coordinates, altitude (eTrex 30; Garmin GmbH), and slope (TruPulse 200/B Laser Rangefinder; Laser Technology Inc.) per vegetation record. Moreover, we measured slope exposure [°], and leaf area index (LAI; LAI‐2200 Plant Canopy Analyzer; LI‐COR Inc.), soil humidity [%] (ThetaProbe ML2x FD probe with Infield7; UMS GmbH), and soil depth [cm] three, five, and 10 times per vegetation record, respectively.

### 2.4 Phenotypic analyses

We analyzed the following traits in situ: plant height (PH) as the shortest distance between ground and the highest flower inflorescence [cm], patch size as a product of maximum length and width [cm^2^], phenology (i.e., flowering or fruiting—when no open flowers were present; flowering status recorded once in both sites at each location within a day), sex (female, hermaphroditic; floral phenotype), and number of flowers per individual (mean number of flowers averaged from three inflorescences). We evaluated phenology as a ratio of fruiting to flowering individuals [%] and sex as the ratio of female to hermaphroditic individuals [%].

In the laboratory, we weighed five fresh leaves per individual, dried these for two to three days at 45°C, and weighed leaves again. We calculated leaf area [mm^2^] using the R package “LeafTraits” vers. 1.0 (Bernhardt‐Römermann, unpubl.). We computed specific leaf areas (SLAs) as a ratio of mean leaf area [mm^2^] and mean leaf dry weight [mg], and leaf dry matter contents (LDMCs) as a ratio of mean leaf dry weight [mg] and mean leaf fresh weight [g] of each individual (Cornelissen et al., 2003; Pérez‐Harguindeguy et al., 2013). We visually estimated trichome density based on upper surface scans of five leaves using three cover categories: *a* ≤ 20% (mean 10%), *b* = 20%–60% (mean 40%), and *c* ≥ 60% (mean 80%). Furthermore, we prepared stomatal imprints from abaxial leaf surfaces of two fresh leaves per individual to measure guard cell length and width [µm] using a 400‐fold magnification (objective 40×, ocular 10×), and to estimate stomatal density [mm^−2^] using a 200‐fold magnification (objective 20×, ocular 10×). Measurements were conducted with an Olympus CH40 microscope. We replicated all stomatal measurements in three randomly chosen areas per leaf and recorded them with the program AxioVision vers. 4.8 (Carl Zeiss Vision International GmbH). Then, we calculated stomatal pore surface (SPS) by guard cell length [µm] × guard cell width [µm] × *π* × 4^–1^ representing the surface area of a widely opened stomatal pore (Balasooriya et al., 2009). We used potential conductance index (PCI) by guard cell length^2^ [mm^2^] × stomatal density [mm^2^] × 10^–4^ as an indicator of stomatal conductance (Holland & Richardson, 2009).

In our study, we compiled appropriate traits to study environmentally related responses of populations. Plant height (PH) and probably also patch size in *T. praecox* relate to competitive ability (Cornelissen et al., 2003; Moles et al., 2009; Pérez‐Harguindeguy et al., 2013). Specific leaf area (SLA) is a correlate of growth rate, and leaf dry matter content (LDMC) indicates leaf robustness and physical resistance (Cornelissen et al., 2003; Pérez‐Harguindeguy et al., 2013). PCI (potential conductance index) is a measure of water‐use efficiency (Holland & Richardson, 2009). Phenology, floral phenotype, and reproductive fitness also strongly depend on environmental conditions, for example, light intensity, temperature, and water availability (Dommée & Jacquard, 1985; König et al., 2018; Manicacci et al., 1996).

### 2.5 Population genetic analyses

We extracted DNA from about 25 mg dry leaf material using the peqGold Plant DNA Mini Kit (PEQLAB Biotechnologie GmbH) and checked the quality electrophoretically. We applied six variable microsatellite loci to infer genetic diversity and differentiation of *T. praecox* populations (C405, D257, D346, D347, E070, and E089; Landergott, Naciri, Schneller, & Holderegger, 2006). To increase PCR yield, we carried out reactions based on the protocol of Landergott et al. (2006) with some modifications: We set up PCRs in final volumes of 20 µl containing 1× *Taq* buffer (Fermentas), 2.5 mM MgCl_2_, 0.2 mM dNTPs, 0.5 µM M13(‐21) tailed forward primer, 1 µM reverse primer, 1 µM M13(‐21) primer, PVP in locus‐specific concentrations (D346, D347, and E089: 0.025%; D257 and E070: 0.05%; C405: 0.1%), 0.025 µg/µl BSA, 5% DMSO, and 0.0125 U/µl *Taq* polymerase (Fermentas). We added PVP to prevent inhibitory effects of polyphenols and used DMSO and BSA to improve the amplification of GC‐rich DNA sequences (Chakrabarti & Schutt, 2001; Farell & Alexandre, 2012; Koonjul, Brandt, Farrant, & Lindsey, 1999). Forward primers were M13(‐21)‐tailed, and we applied differently labeled M13(‐21) primers for amplifications to save cost and time (Schuelke, 2000). We labeled loci C405, D347, and D257 with M13(‐21) IRD 700, and loci D346, E070, and E089 with M13(‐21) IRD 800. We multiplexed C405 and D346, D347, and E089, and E070 and D257.

Then, we applied locus‐specific touchdown programs (D346, E070, and E089: 52–42°C; C405 and D347: 57–46°C; D257: 65–53°C) to increase PCR specificity (Korbie & Mattick, 2008). Compared with the protocol of Landergott et al. (2006), we decreased annealing temperature to 50°C at locus D347 to improve PCR quality. Cycling was as follows: initialization at 94°C for 5 min; *n* × touchdown cycles with previous denaturation at 94°C for 30 s, annealing at touchdown specific *t*_a_ starting at *t*_max_ to a final temperature *t*_min_ with a reduction of 1°C in each cycle, and elongation at 72°C for 30 s; 20 × main amplification cycles of denaturation at 94°C for 30 s, annealing at locus‐specific *t*_a_ for 90 s, and elongation at 72°C for 35 s; 10 × M13‐specific cycles of denaturation at 94°C for 30 s, annealing at 53°C for 45 s, and elongation at 72°C for 60 s; final elongation at 72°C for 10 min; and sample storage at 4°C. We combined products of different labels (IRD 700, IRD 800) for fragment length analyses using a LI‐COR Long Readir 4200 (Global Edition IR2 DNA Sequencer; LI‐COR Inc.). Fragments were analyzed visually using an internal size standard.

### 2.6 Statistical analyses

We analyzed environmental, phenotypic and genetic data for each location (Ka, Bu, Mu, and Wa), for badlands and grassland sites (B and G), and for local badlands and grassland sites (local sites: KaB, KaG, BuB, …, and WaG; see Table 1). We calculated means for numerical variables and medians for ordinal variables. Environmental calculations are based on vegetation records, and calculations concerning phenotype and genotype are based on individuals.

### 2.7 Environmental and phenotypic analyses

We statistically evaluated data with R vers. 3.5.2 (R Core Team, 2018). In each location, species richness (S) was calculated with the R package “vegan” vers. 2.5‐3 (Oksanen et al., 2018). We generated community‐weighted Ellenberg indicator values (wIV; indicator values published in Ellenberg, Weber, Düll, Wirth, & Werner, 2001) of light availability (wL), climatic continentality (wK), soil humidity (wF), temperature (wT), soil reaction (wR), and soil fertility (wN) following the formula wIV = ΣIV*_i_* * *p_i_* (*p_i_* = frequency of species *i* in a vegetation record). We excluded wL, wK, wF, and wN because these indicator values showed almost no differences among location, between sites and between local sites (data not shown).

First, we examined normal distribution between groups of environmental factors and phenotypic traits by a combined approach consisting of Shapiro–Wilk tests called with the function shapiro.test() and *Q*–*Q* plots called with qqnorm(). If Shapiro–Wilk test results were scarce above or below the significance threshold (*p* ~ .05), we used *Q*–*Q* plots to verify/falsify normal distribution. Second, we tested differences between the two groups with unpaired, two‐sided Student's *t* test (*t*, Welch implementation for unequal variances) by t.test() or with Mann–Whitney *U* test (W) by wilcox.test() if data were non‐normally distributed. For data sets with more than two groups, we applied analyses of variance (*F*, ANOVA) by aov() or Kruskal–Wallis tests (chi‐square, *H* test) by kruskal.test() if data were non‐normally distributed. For ANOVA, we checked with the Fligner–Killeen test by fligner.test() whether variances in each of the groups were homogeneous. We evaluated statistical differences at group levels with Tukey's “honest significant difference” method using the function TukeyHSD() if ANOVA was applied, or pairwise Wilcoxon rank‐sum test with Holm correction using the function pairwise.wilcox.test() if the Kruskal–Wallis test was applied. Phenology and floral phenotype are nominal data, and thus, we performed Pearson's chi‐squared test with Yates' continuity correction using the function cisq.test() to examine differences among locations, between sites, and between local sites.

Since standard statistical tests do not allow statements on whether environmental and phenotypic differences are strongest among locations, between sites, or between local sites (site × location), we carried out multiple linear regression models (LMs) implemented in the function lm(). We log‐transformed response variables for normality and treated Ellenberg indicator values as numeric. We also executed multiple generalized linear models (GLMs) for count data with Poisson error structure, and proportion and binomial data with binomial error structure by the function glm() (Crawley, 2015).

In general, we found location‐wise environmental and phenotypic differences to be the smallest (see Tables S2 and S4). To examine sites regardless of location effects, we additionally calculated linear mixed effect models (LME) and generalized linear mixed effect models (GLMM) between environmental/phenotypic variables and sites with locations as a random factor using functions lme() and glmer() implemented in the R packages “nlme” vers. 3.1‐137 and “lme4” vers. 1.1‐20 (Bates, Mächler, Bolker, & Walker, 2015; Pinheiro, Bates, DebRoy, & Sarkar, 2018). For LMEs, we log‐transformed continuous response variables to achieve normality, and for GLMMs, we again used the same error structures for response variables as in GLMs. Model simplification was performed with backward selection applying the function update(): The least significant variable (at least *p* > .1) was excluded until the final minimal adequate model was reached (Crawley, 2015). Then, we calculated ANOVAs with the function aov() to justify each model simplification step. If the variable selection was ambiguous (e.g., when some levels within locations and local sites both marginal significant), we calculated the Akaike information criterion (AIC) value using AIC() and chose the model with the lowest AIC value for further model simplification steps. We checked model assumptions for normality, homoscedasticity, linearity, and outliers with the R function plot(). In general, LME/GLMM results supported the results of standard statistical tests indicating negligible local effects (see Tables S1 and S3). Therefore, results and inferences of environmental/phenotypic site differences are based on standard statistical tests.

Moreover, we ran a detrended correspondence analysis (DCA) with means of phenotypic traits based on local site data applying the function decorana() implemented in the R package “vegan” (Oksanen et al., 2018). Because the length of the first DCA axis was lower than 2.5, we conducted a principal component analysis (PCA; Hotelling, 1933; Pearson, 1901) with prcomp() using the previous data set. Then, we correlated standardized continuous and categorical environmental factors (local site data) with PCA axes using envfit() based on 1,000 permutations and displayed only significant (*p* < .05) correlations. We excluded slope exposure because local sites were always situated on the same slope exhibiting no differences in exposure (see Figure 1). PCA based on individual phenotypic trait data revealed similar patterns, but we only illustrated PCA based on local site‐wise means for reasons of clarity and comprehensibility.

### 2.8 Population genetic analyses

We performed population genetic analyses including all individuals that are characterized by at least three microsatellite loci. Filtering procedure results in a final sample size of 109 individuals (Table 1). Locus coverage was 85% per individual, that is, on average, 85% of loci were present in an individual (about five loci per sample on average; see Table S6 and data repository). Locus coverage was comparable and not significantly different between sites (86% in badlands and 84% in grasslands; *W* = 1532, *p* = .62) and local sites (*χ* ^2^ = 9.73, *p* = .20). Moreover, genetic diversity indices only slightly differed among loci (see Table S7). Therefore, locus coverage and locus diversity did not bias genetic differentiation patterns. As a consequence of species' tetraploid nature, data analyses were challenging due to the unknown allele dosage of partial heterozygotes. We used a combined approach (see, e.g., Wiehle & Prinz et al., 2014), by transforming present alleles in a binary matrix to analyze the data in a dominant manner, and by estimating allele frequencies of partial heterozygotes in a codominant manner for population genetic indices based on Hardy–Weinberg equilibrium (Hardy, 1908; Weinberg, 1908).

Initially, we transformed individual fragment sizes in a presence/absence matrix. Next, we used GenAlEx vers. 6.501 (Peakall & Smouse, 2006, 2012) to compute multilocus mean values for allelic richness *N*_A_, private alleles *P*_AP_ [%], and Nei's unbiased genetic diversity *uh* (Nei, 1978) correcting for different sample sizes between groups. We ran AMOVAs (analysis of molecular variance; Excoffier, Smouse, & Quattro, 1992) to calculate hierarchical partitioning of variation between and within groups. In addition, we performed two‐dimensional principal coordinate analyses (PCoAs; Gower, 1966) based on Nei's genetic distances (Nei, 1978) via a covariance matrix with data standardization and illustrated the results in Excel 2016 (Microsoft). Considering the results of PCoA, we split the badlands site at location Burg Gleichen (Bu) into three subpopulations that better represent substructure (Figure 7), and repeated AMOVA. We also examined PCAs for identical axes scores. We found no overlapping individual points (Figures 5, 6, 7), and thus no evidence for clonal ramets. We tested isolation by distance among local sites with the Mantel test (Mantel, 1967).

In the second part, we conducted analyses in STRUCTURE vers. 2.3.4 (Pritchard, Stephens, & Donnelly, 2000) based on locus‐wise individual fragment sizes to infer population genetic structure. We were not able to assign multilocus microsatellite genotypes and to test precisely for linkage disequilibrium due to unknown allele dosages in allotetraploid species. Microsatellite loci were therefore assumed to be unlinked in STRUCTURE analyses. Moreover, populations should be in a panmictic state, which is also hard to assess with unknown allele dosages. The sexual pathway allows selfing in *T. praecox*, but location‐wise estimated population size was quite high (individuals per vegetation record × area of location; Ka: 44,000, Bu: 91,000, Mu: 4,700, Wa: 13,000) and flowers were frequently visited by pollinators possibly allowing sufficient mating processes. Furthermore, genetic diversity was not reduced in badlands sites (see Table 1) suggesting no remarkable effects of selfing. Thus, populations in local sites are probably in a panmictic state. We set a burn‐in of 10,000 and a MCMC of 50,000 replicates and ran a locprior model because of the expected weak genetic structure among genetic groups. Settings were replicated 10 times for each value of *K* (*K* = number of genetic clusters). We chose a range of *K* from one to eight, and the optimal *K* was determined by STRUCTURE HARVESTER (Earl & vonHoldt, 2012) using the Evanno method with delta *K* values. Replicates of optimal *K* were merged with CLUMPP vers. 1.1.2 (Jakobsson & Rosenberg, 2007) and plotted with DISTRUCT vers. 1.1 (Rosenberg, 2004).

In the third part of the analysis, we used the R package “polysat” vers. 1.7‐4 (Clark & Jasieniuk, 2011; Clark & Schreier, 2017) to calculate location‐, site‐, and local site‐wise expected heterozygosity *H*_e_ (Nei, 1973) and genetic differentiation *G*_ST_ (Nei, 1986) values. Within R, we created *.txt files in Tetrasat format (Markwith, Stewart, & Dyer, 2006) with cat(), transformed the *.txt files in genambig files with read.Tetrasat(), and ran the function simpleFreq() to calculate allele frequencies. This function assumes that in partially heterozygous genotypes, “all alleles have an equal chance of being present in more than one copy” (Clark, 2019). We calculated H_e_ per population transforming simpleFreq() output into *.genpop files with freq.to.genpop() and using Hs() function implemented in the R package “adegenet” vers. 2.1.1 (Jombart, 2008; Jombart & Ahmed, 2011; Jombart et al., 2018). *G*_ST_ values were generated based on simpleFreq() output with calcPopDiff() setting 10,000 bootstrap replicates. Then, we performed Pearson or Spearman (if non‐normally distributed data) correlation tests with the function cor.test() between genetic diversity indices and sample size.

Testing for loci under selection in polyploid species remains still problematic because programs have to deal with unknown allele dosage in partial heterozygotes to calculate *F*_ST_/*G*_ST_ values. Results revealed the strongest genetic differentiation between local sites, and therefore, *H*_e_ values of local sites were chosen for loci outlier analysis. Based on *H*_e_ values, we performed the ln RH test (Soto‐Cerda & Cloutier, 2013) to check for outlier microsatellite loci. Natural logarithm (ln) ratio of gene diversity [(1/(1 − *H*_e(pop1)_))^2^ − 1]/[(1/(1 − *H*_e(pop2)_))^2^ − 1] was calculated for each group combination, and ln RH estimates were standardized to zero mean and standard deviation of one (Kauer, Dieringer, & Schlötterer, 2003; Schlötterer, 2000). Ninety‐five percent of the neutral loci are expected between −1.96 and 1.96, and ln RH values outside this range were considered as outliers (Soto‐Cerda & Cloutier, 2013).

## 3 RESULTS

### 3.1 Environmental and phenotypic differentiation

**Figure 2:**
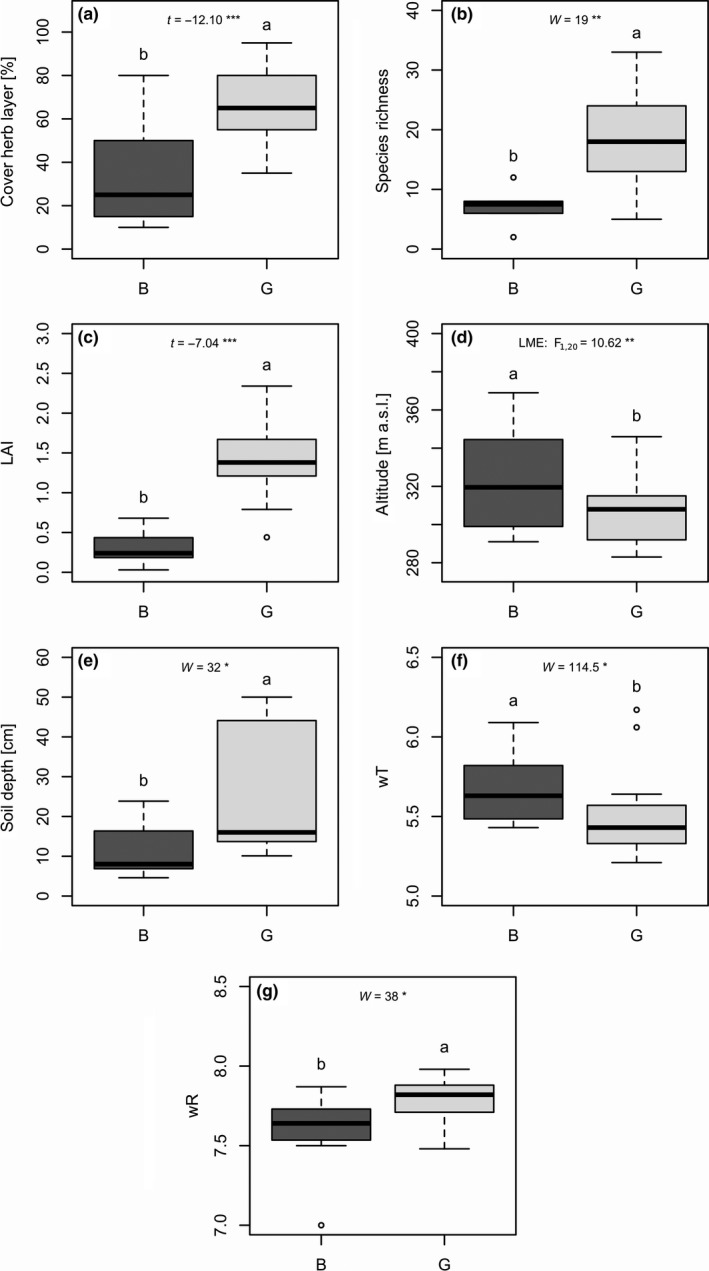
Boxplots of (a) cover of herb layer, (b) species richness, (c) leaf area index (LAI), (d) altitude, (e) soil depth and community‐weighted Ellenberg indicator values of (f) temperature (wT), and (g) soil reaction (wR) demonstrate significant environmental differences between badlands (B; dark gray) and grassland (G; light gray) sites including all locations. Measurements are based on 25 vegetation records. See Table S1 for detailed statistics. Significance levels: ***p < .001, **p < .01, *p < .05

Among locations, we observed (marginal) significant differences (*p* < .1) in slope, slope exposure, altitude, and soil depth (see Table S1). The slope was in general moderate (16.0–29.2°), but steepest at Mu. Locations were mostly south‐ to west‐exposed and differed slightly in altitude (292–350 m) with Wa being the highest location. We detected remarkably deeper soils in Mu and Wa (27.4 cm, 34.0 cm) than in Ka and Bu (11.0 cm, 10.3 cm). In badlands, we detected significantly lower cover of herb layer, species richness, LAI, soil depth, and wR, and higher wT compared with grasslands (Figure 2). LME and GLMM results were comparable to differences between sites ascertained by t and W tests but revealed additionally a significant positive relationship between badlands sites and altitude (see Table S1). Local sites showed (marginal) significant differences for almost all of the before stated site factors; however, slope (in Wa) and soil moisture (in Mu) were additionally found to be significantly different. LMs and GLMs revealed the strongest differences between sites (cover of herb layer, species richness, LAI, altitude, soil depth, and wR) followed by locations (slope, slope exposure, altitude, and soil depth) and local sites (wR, see Table S2). Thus, sites and local sites showed more (marginal) significant differences (64%) compared with locations.

**Figure 3:**
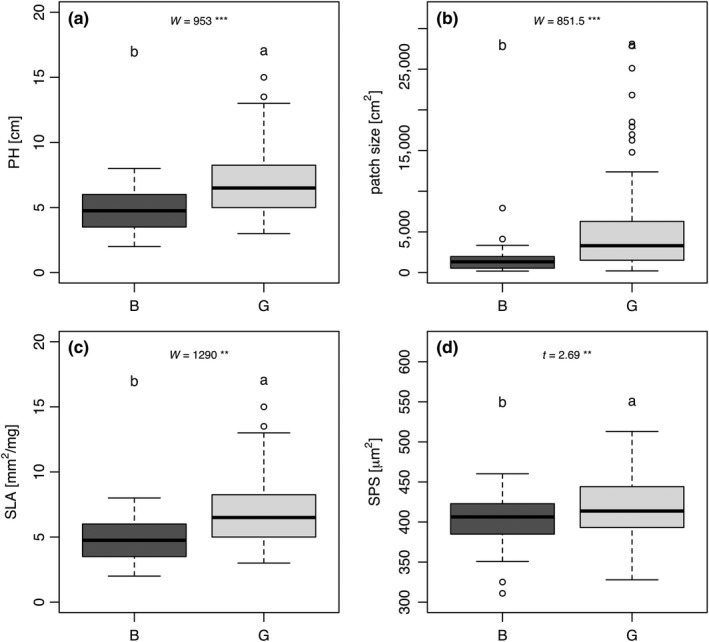
Boxplots of (a) plant height (PH), (b) patch size, (c) specific leaf area (SLA), and (d) stomatal pore surface (SPS, bottom, abaxial leaf surface) show phenotypic traits with significant differences between badlands (B; dark gray) and grassland (G; light gray) sites including all locations. Measurements are based on 125 individuals. See Table S3 for detailed statistics. Significance levels: ***p < .001, *p* < .01**

*Thymus praecox* individuals among locations significantly differed in RH, patch size, mean number of flowers/individual, floral phenotype (percent of female individuals), phenology (percent of fruiting individuals), SLA, and PCI (see Table S3). When comparing badlands with grassland sites, individuals revealed significantly lower RH, patch size, SLA, and SPS (Figure 3; Figures S4, S5). LME and GLMM results were comparable to differences between sites ascertained by t and W tests but revealed, additionally, a positive influence of badlands sites on number of flowers/individual (significant) and on fruiting individuals (marginal significant; see Table S3). Significance of number of flowers/individual within GLMM has to be treated with caution because local sites revealed contrasting effects: Ka and Bu badlands showed (nonsignificant) more number of flowers/individual, and Mu and Wa badlands showed significantly fewer number of flowers/individual. In general, we detected similar differences between local sites and between sites. In LMs and GLMs, we observed the most (marginal) significant differences between sites (RH, patch size, number of flowers/individual, phenology, and SLA) followed by local sites (RH, patch size, number of flowers/individual, and SPS) and locations (PCI, phenology, and floral phenotype; see Table S4). Sites and local sites revealed more significant phenotypic differences (75%) than locations.

**Figure 4:**
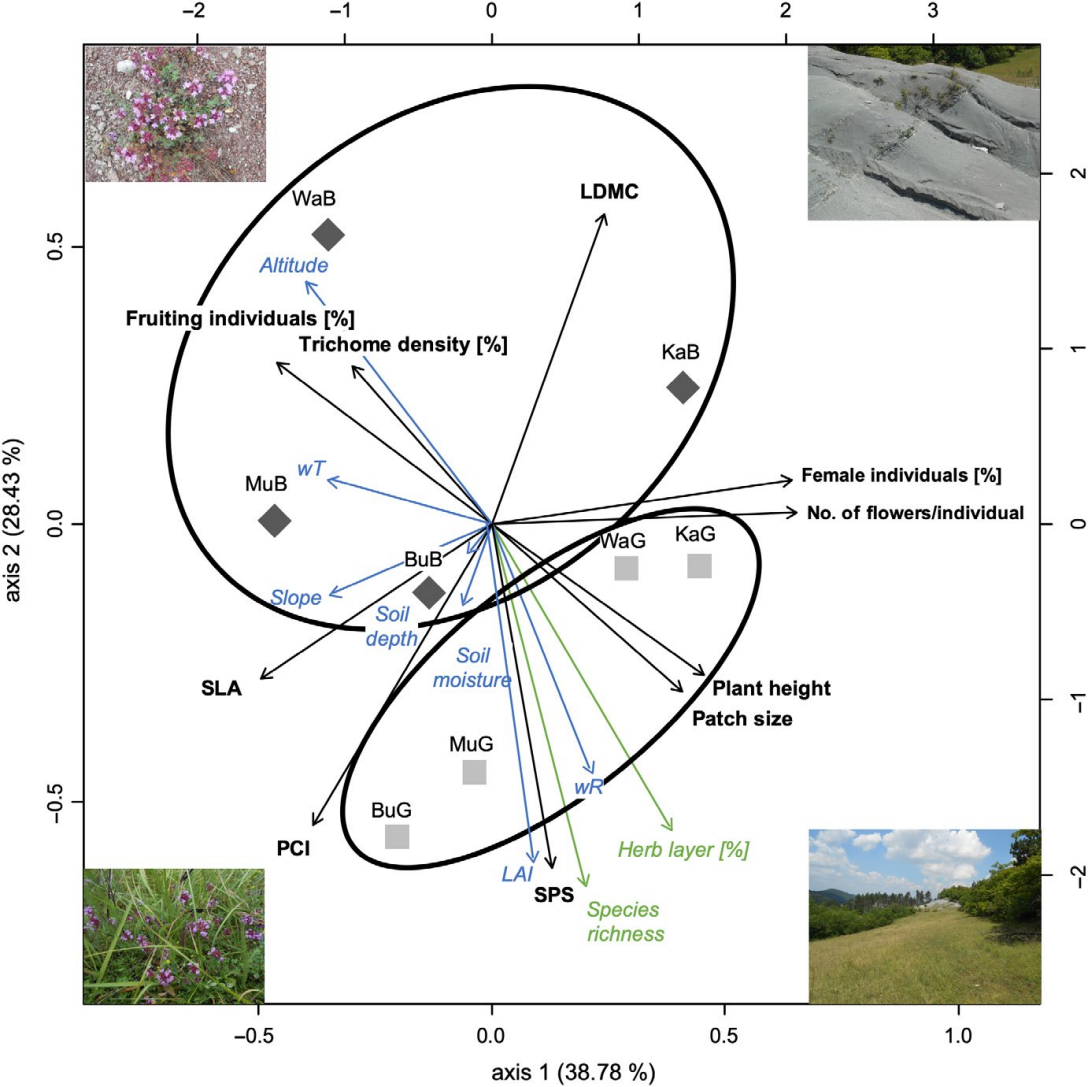
Principal component analysis (PCA) shows phenotypic trait differences between local badlands (dark gray rhombs) and grassland sites (light gray squares) and trait correlations with environmental factors (see Tables S1 and S3 for abbreviations). The first two axes explain 67% of trait variation. Only significant (*p* < .05) environmental factors are illustrated. Plant phenotypic traits are marked in bold and environmental factors in italics

The PCA of mean phenotypic traits indicated badlands (B) and grassland (G) sites as separate groups (Figure 4). The first two PCA axes explained 67% of the total variation. Sites are mainly separated by the second PCA coordinate representing 28% of the variation. Badlands individuals exhibited increased LDMC values, higher leaf trichome density, and earlier phenology, correlating positively with altitude and wT. Lower RH, patch size, SPS, and PCI values of badlands compared with grassland individuals are associated with decreasing LAI, wR, soil moisture (and soil depth), species richness, and cover of herb layer. Percent of female individuals, mean number of flowers/individual, and SLA correlate with slope, but these traits are rather responsible for the separation of sites along the first PCA axis.

### 3.2 Genetic diversity and differentiation

Genetic diversity indices of *T. praecox* differed only slightly among locations, sites, and local sites, but values were in general relatively high (Table 1). Among locations, Wa exhibited the highest values of genetic diversity indices, whereas Bu showed the lowest ones. Between sites, genetic diversity was scarcely lower in badlands than in grasslands concerning *N*_A_, *P*_Ap_, *H*_e,_ and *uh*. Local sites showed no general tendency in genetic diversity indices. Sample size significantly positively affected *N*_A_ (*S* = 31.8, *r*_SP_ = 0.93, *p* < .001) and *H*_e_ (*S* = 192.3, *r*_SP_ = 0.58, *p* < .05), but not *P*_Ap_ (*S* = 398.7, *r*_SP_ = 0.12, *p* = .67) and *uh* (*S* = 285.2, *r*_SP_ = 0.37, *p* = .19).

**Figure 5:**
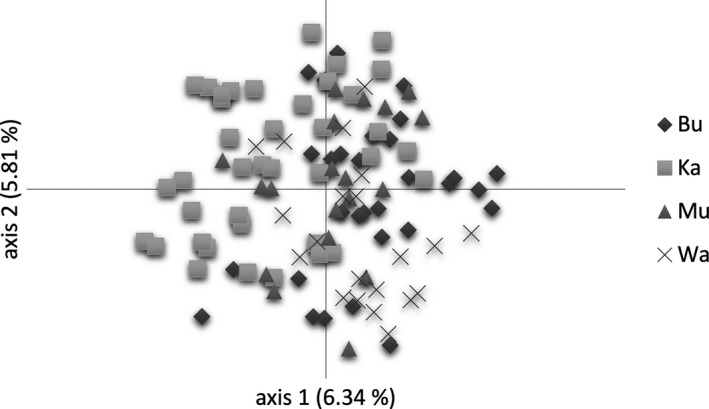
Principal coordinate analysis (PCoA) based on Nei's genetic distance and 109 *Thymus praecox* individuals from locations Kallenberg (Ka), Burg Gleichen (Bu), Mühlburg (Mu), and Wachsenburg (Wa). Both axes represent about 12% of the total genetic variation

**Figure 6:**
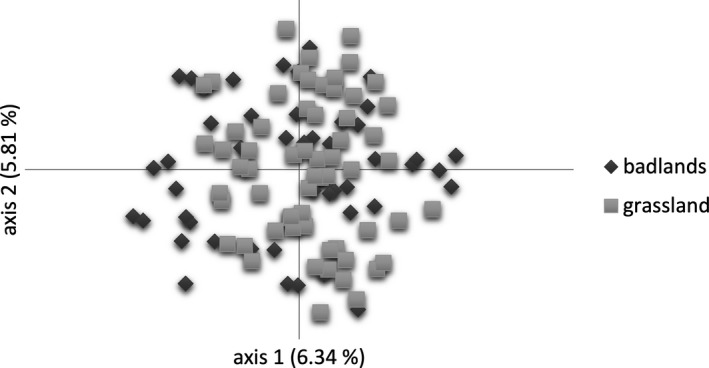
Principal coordinate analysis (PCoA) based on Nei's genetic distance and 109 *Thymus praecox* individuals from badlands (dark gray rhombuses) and grassland (light gray squares) sites. Both axes represent about 12% of the total genetic variation

**Figure 7:**
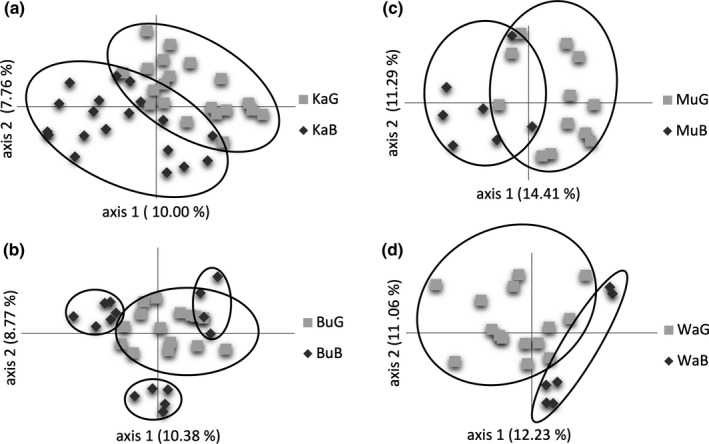
Principal coordinate analyses (PCoAs) based on Nei's genetic distance and 109 *Thymus praecox* individuals from local badlands (dark gray rhombuses) and grassland (light gray squares) sites at locations (a) Kallenberg (Ka), (b) Burg Gleichen (Bu), (c) Mühlburg (Mu), and d) Wachsenburg (Wa). Both axes represent about 18%, 19%, 26%, and 23% of the total genetic variation, respectively

**Figure 8:**
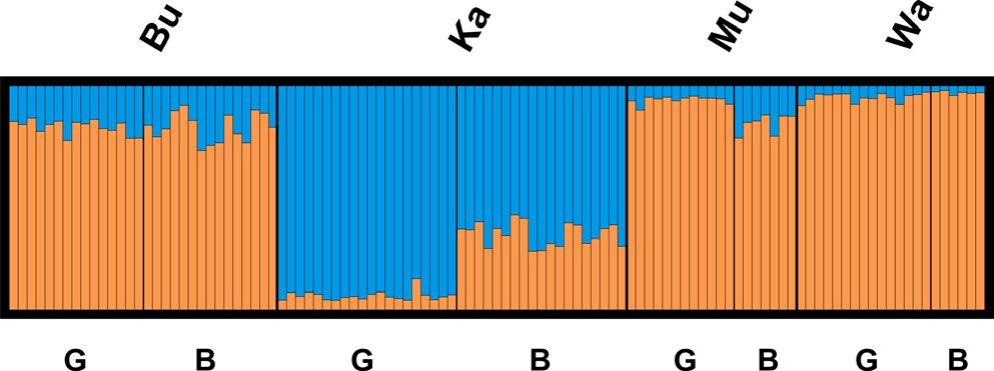
Population genetic structure inferred from the likeliest *K* value (*K* = 2, ∆*K* = 13.42) based on 109 *Thymus praecox* individuals. Each bar represents one individual, and different sites within each location are indicated by B (badlands) and G (grassland). Bu, Burg Gleichen; Ka, Kallenberg; Mu, Mühlburg; Wa, Wachsenburg

Main genetic variation occurred within locations, sites, and local sites having 86%–99% (Table 2). We observed a significant hierarchical partitioning of variation with 95% of variation within and 5% of variation among locations (Φ = 0.045, *p* < .001), and 99% and 1% variation between sites (Φ = 0.011, *p* = .01). PCoAs indicated a complete intermixture of individuals among locations and between sites (Figures 5 and 6). The first two PCoA axes explained 12% of the total genetic variation. We detected significantly higher genetic variation by 8% (Ka), 14% (Bu), 10% (Mu), and 10% (Wa) among local badlands and grassland sites (Table 2). PCoAs of local sites explained 18%–26% of total genetic variation, and individuals were well‐separated by sites (Figure 7). Genetic differentiation patterns are supported by lowest genetic differentiation between sites (*G*_ST_ = 0.003) and locations (*G*_ST_ = 0.015), and highest genetic differentiation between local sites (*G*_ST_ = 0.035). We found weak, but significant, isolation by distance among local sites (*R_xy_* = 0.109, *p* = .01, see Figure S6). STRUCTURE analysis of individuals is resolved by *K* = 2 genetic clusters (∆*K* = 13.42), and individuals are composed of different amounts of each genetic cluster (Figure 8). The genetic structure indicates at least for local genetic differentiation between sites at Ka and Mu. Natural logarithm RH microsatellite loci outlier analysis showed up no values beyond the range of −1.96 to 1.96 and hence indicated for no SSR outlier loci under selection (see Table S5).

**Table 2:** Analyses of molecular variance (AMOVA) among Kallenberg (Ka), Burg Gleichen (Bu), Mühlburg (Mu), and Wachsenburg (Wa), between badlands and grassland sites (B and G) and between local sites (Location + Site) based on 109 *Thymus praecox* individuals

	Group	*df*	Sum of squares	Est. variance	Variation [%]	Φ Statistics
Location	Among groups	3	69.86	0.49	**5**	**0.045, *p* = .001**
	Within populations	105	1,084.36	10.33	**95**	
	Total	108	1,154.22	10.82		
Site	Among groups	1	17.04	0.12	**1**	**0.011, *p* = .01**
	Within populations	107	1,137.19	10.63	**99**	
	Total	108	1,154.22	10.75		
Location + Site	Among groups	7	149.39	0.85	**8**	**0.079, *p* = .001**
	Within populations	101	1,004.83	9.95	**92**	
	Total	108	1,154.22	10.80		
Ka	Among groups	1	27.32	0.89	**8**	**0.082, *p* = .001**
	Within populations	37	369.45	9.99	**92**	
	Total	38	396.77	10.88		
Bu	Among groups	1	3	56.58	**14**	**0.144, *p* = .001**
	Within populations	28	26	231.65	**86**	
	Total	29	29	288.23		
Mu	Among groups	1	19.27	1.06	**10**	**0.097, *p* = .01**
	Within populations	17	168.42	9.91	**90**	
	Total	18	187.68	10.97		
Wa	Among groups	1	19.65	1.11	**10**	**0.098, *p* = .001**
	Within populations	19	193.40	10.18	**90**	
	Total	20	213.05	11.28		

Note

Significant results are indicated in bold.

Abbreviations: *df*, degrees of freedom; est. variance, estimated variance.

## 4 DISCUSSION

Results indicate small‐scale phenotypic (morphological, functional) and genetic differentiation in *T. praecox* confirming our hypothesis of site‐dependent patterns due to different microhabitat conditions. This study is in line with a broad range of literature showing phenotypic plant trait responses and genetic differentiation patterns to varying abiotic and biotic environmental conditions (e.g., Bucher et al., 2016; Karbstein et al., submitted; König et al., 2018; Linhardt & Grant, 1996; Odat et al., 2004). In *T. praecox*, differentiation is probably promoted by site‐dependent microhabitat differences mainly in light, temperature, and soil conditions. Environmental and phenotypic differences are pronounced between sites, whereas genetic differentiation was strongest and only significantly present between local sites. For the first time, we were thus able to detect both detailed plant intraspecific phenotypic and genetic site differentiation between badlands and surrounding grasslands.

In general, we observed congruent environmental and phenotypic patterns exhibiting the strongest differentiation between sites and local sites. Despite the strongest site differentiation, many plant phenotypic traits also differed among locations presumably caused by local environmental effects (weak differences in altitude, slope exposure, slope, and soil depth). For example, shallow, south‐exposed locations compared with flat, west‐exposed ones have a tenser hydrological balance that may influence phenotypic trait expression (e.g., Ka compared with Mu). However, badlands are notably different from surrounding grasslands due to lower vegetation cover and species diversity, higher light availability, steeper slopes, shallower, drier and less calcareous soils, and slightly higher temperatures (wT). Badlands with steeper hill slopes are mostly situated above grasslands, which explains significant higher altitudes in statistical analyses (see Figure 1). Due to an absence of soil organic matter content, less soil fertility can also be assumed in badlands sites. In Northeastern Spain, the fertility of badlands slopes was also found to be very low due to an absence of soil organic matter content (Fairbridge, 1968; Guàrdia, 1995; Navas, Machín, & Navas, 1999).

*Thymus praecox* individuals in badlands indicate morphological and functional trait responses to enhanced light, temperature, drought, and nutrient stress. They are shorter in height and smaller in patch size as a response to increased light intensity, higher temperatures (wT), and weakly to lower soil humidity (PCA). Increased light intensity is able to reduce plant elongation and growth, and thus plant height (Gratani, 2014; Jansen, 2002) of *T. praecox*. Plant height is also an important determinant of species ability to compete for light (Díaz et al., 2016; Moles et al., 2009). However, biotic competition for light in badlands seems to play a negligible role in comparison with abiotic light stress. Moreover, drought stress is potentially one of the most important constraints limiting nutrient uptake, photosynthesis, and thus plant growth (Farooq, Wahid, Fujita, & Basra, 2009; Jaleel et al., 2009).

Specific leaf area (SLA) relates to photosynthesis and relative growth rate (Feng, Fu, & Zheng, 2008; Garnier, 1992; Pérez‐Harguindeguy et al., 2013), and thus, lower SLAs in badlands indicate decreased relative growth rates. SLA varies throughout the year and tends to decrease toward the end of the growing season (Römermann, Bucher, Hahn, & Bernhardt‐Römermann, 2016). The herein measured SLA is therefore only a snapshot within the growing season of *T. praecox* although an intraspecific relationship between plant height and SLA is reasonable at the beginning or middle of the growing season. In contrast to plant height, SLA of *T. praecox* correlates more with slope, soil depth, and moisture instead of light availability (see also Garnier et al., 2004; Poorter & De Jong, 1999; Reich et al., 1999). Less water and nutrient availability in badlands probably negatively influence relative growth rates. Furthermore, low‐SLA and high‐LDMC leaves (inverse relationships) reflect thicker palisade parenchyma, epidermal tissue, and leaf robustness probably caused by higher light intensity (Evans, 1999; Gratani, 2014; Pérez‐Harguindeguy et al., 2013; Ryser & Urbas, 2000; Scheepens, Frei, & Stöcklin, 2010). Badlands habitats are wind‐exposed and characterized by steady erosion (Klug, 2010; Werneburg, 2003), and more robust leaves may be advantageous for persisting there. Increased leaf thickness can also regulate transpirational loss (Farooq et al., 2009).

We also observed smaller stomata (SPS), and in PCA, less stomatal conductance (PCI) of individuals growing in badlands sites associated with higher light intensity, sparser vegetation cover, and lower soil moisture. Stomatal density increase and stomatal size decrease with increasing light intensity resulting in a bigger membrane surface‐to‐volume ratio and allowing a faster conductance regulation (Casson & Gray, 2008; Drake, Froend, & Franks, 2013). A reduced conductance is often reported as an adaptive response in plants growing under drought stress conditions (Spence, Wu, Sharpe, & Clark, 1986; Xu & Zhou, 2008). Light and drought stress, therefore, influences stomatal conductance, but both factors are only hard to distinguish. Accordingly, leaf trichome density that is positively related to temperature (wT) is slightly increased in almost all badlands sites also suggesting a transpirational regulation (Bañon et al., 2004; Quarrie & Jones, 1977).

Reproductive fitness derived from mean number of flowers/individual exhibits indifferent patterns between local sites. Reproductive fitness is decreased only in some badlands (Mu, Wa). An explanation might be the deficient soil moisture and higher temperature (wT) reducing the investment of individuals in reproduction. Studies on *T. vulgaris* reported female individuals with smaller flowers and remarkably higher seed set than gynodioecious ones (Dommée & Jacquard, 1985; Manicacci et al., 1996; Thompson et al., 2002). In Wa, grasslands have higher percentages of female individuals compared with badlands probably also explaining fewer number of flowers/individual, seed set, and reproductive fitness. However, we observed a slightly increased amount of hermaphrodites compared with females in badlands. Previous studies usually recorded more females in disturbed environments providing higher seed production and therefore higher colonization rates (Dommée & Jacquard, 1985; Manicacci et al., 1996). Nevertheless, hermaphroditic individuals can ensure reproduction by selfing if pollination vectors (e.g., bees, bumblebees, hoverflies, and beetles) infrequently fly on badlands due their unfavorable light and temperature conditions (see Corbet et al., 1993; Lundberg, 1980; Papanikolaou, Kühn, Frenzel, & Schweiger, 2017). Intriguingly, Ehlers and Thompson (2004) examined hermaphroditic individuals of *T. vulgaris* that flower earlier, but for a longer time period. On the one hand, slightly earlier flowering of *T. praecox* individuals is explainable by the fact of more hermaphrodite individuals in badlands. On the other hand, slight precocity in phenology can also be explained by higher local temperatures of badlands due to the heating of bare, vegetation‐poor stones, and high‐temperature fluctuations in spring and summer (see, e.g., Menzel et al., 2006).

Genetic differentiation and structure of *T. praecox* were generally low, but statistically significantly different among locations, between sites, and between local sites (*G*_ST_: 0.003–0.035; AMOVA: 1%–14%; Tables 1 and 2; Figure 8). Local sites revealed a more than three times stronger genetic differentiation than sites or locations. This pattern is astonishing because local sites were only separated by a few meters and locations by several kilometers. Consequently, results strongly indicate locally differentiated genotypes. The observed site‐differentiation patterns are supported by literature investigating genetic differentiation due to (micro)habitat differences (e.g., Ali et al., 2012; Linhardt & Grant, 1996; Odat et al., 2004; Sakaguchi et al., 2018). In general, we observed a remarkable phenotypic and genetic differentiation between local sites suggesting associated small‐scale differentiation patterns. Low sample sizes at badlands in Mu and Wa may limit the explanatory power of results, but differentiation patterns were comparable to those observed for the other locations (Ka, Bu). Additionally, small microsatellite marker size may also influence the strength of observed genetic differentiation patterns.

Different factors explain the unveiled genetic differentiation. Natural selection acts on genotypes (Dawkins, 2008, 2010) and is thus able to influence genetic differentiation patterns in *T. praecox*. Differentiation caused by abiotic factors tends to be sharper than differentiation caused by biotic ones (Epperson, 1995; Linhardt & Grant, 1996; Linhart, Snyder, & Gibson, 1994) supporting the patterns observed in PCoAs. Abiotic factors vary gradually or abruptly, whereas biotic factors change more dynamically (e.g., competitors, herbivores, or pollinators; Linhardt & Grant, 1996). Biotic factors play perhaps a negligible role in shaping genetic differentiation pattern due to almost absent vegetation on badlands reducing intra‐ and interspecific competition. Several abiotic factors probably promote genetic differentiation of *T. praecox* individuals in badlands: high solar radiation, huge temperature fluctuations especially in summer, and low soil moisture resulting from shallow soils and steep slopes. Natural selection may act on the available genetic variation in *T. praecox* forming differentiated, potentially adapted genotypes between sites. Genetic diversity was generally high (*H*_e_: 0.832–0.870; compared with Pairon, Jacquemart, & Potter, 2008) as expected from an allotetraploid species. High genetic variation provides the basis for phenotypic plasticity and evolutionary processes such as local adaptation (Comai, 2005; Gratani, 2014). Minor differences in genetic diversity among locations and sites are probably caused by different sample sizes correlating positively with allelic richness and expected heterozygosity in our study.

Most settlements in the Drei Gleichen region were founded in the eighth‐ to ninth‐century AD (e.g., Mühlberg as the oldest documented settlement in Thuringia 704 AD), and badlands may have been formed since the medieval ages by sheep‐ and goat‐grazing (Bricks, 1993; Klug, 2006, 2010; Kräham, 2002). Selection due to abiotic conditions could have acted since 500 to 1,200 years, a period that is sufficient for selection processes despite moderate selective pressures (see, e.g., Daehler, Anttila, Ayres, Strong, & Bailey, 1999; Jain & Bradshaw, 1966; Linhardt & Grant, 1996).

Limited gene flow is probably the most important factor enabling the genetic differentiation of *T. praecox* individuals between local sites (see Linhardt & Grant, 1996). Also when assuming strong selective forces in local badlands sites, restricted gene flow is needed to stabilize sharp differentiation patterns. Otherwise, we would have observed significantly overlapping genotypic patterns between local sites, which is not the case in this study. Bees and bumblebees mainly pollinate *T. praecox*, and their activity may be hampered in badlands due to high light intensity and temperatures during spring and summer months (Corbet et al., 1993; Lundberg, 1980; Papanikolaou et al., 2017). Particularly during flowering time, pollination may be limited. Insects usually visit flowers when days are dry and sunny. Solar radiation rapidly heats up badlands areas (>60°C observed above the soil) probably reducing pollinator activity. This potentially lowers gene flow between sites also contributing to relatively high percentages of genetic variation between local sites (5%–14%, Table 2), despite very low distances between them (1–20 m). Furthermore, badlands and grasslands at Bu are more mixed in comparison with other locations (see Figure 1). The rather patchy distribution of these sites probably causes varying gene flow and explains genetic subgrouping of badlands individuals at this location. Furthermore, in a sympatric situation, divergence in flowering time can isolate populations (Baack, Melo, Rieseberg, & Ortiz‐Barrientos, 2015). Slightly earlier flowering of badlands individuals is in accordance with the theory stating that before microclimatic conditions become worse in open habitats, earlier flowering can ensure the reproductive success of individuals (Sakaguchi et al., 2018). Furthermore, self‐compatible flowers, even when selfing occurs in low frequencies, guarantee the reproduction of badlands individuals that are less‐visited by pollinators promoting genetic differentiation among sites, besides limited gene flow as well.

However, sites did not exhibit genetically differentiated groups in general, and genotypes are completely mixed (Figure 6). An explanation for this observation might be the applied marker type. Microsatellites were used to explain genetic differentiation among *T. praecox* individuals, and they are widely assumed to be selectively neutral when occurring in noncoding regions (Ellegren, 2004; Vieira, Santini, Diniz, & Munhoz, 2016). Landergott et al. (2006) provided no information about the intronic or exonic affiliation of developed microsatellites, and we did not find signals of microsatellite loci under selection as indicated by our ln RH outlier analysis. The applied microsatellite markers are dominated by a two‐nucleotide motif which is potentially more selectively neutral compared with trinucleotide motifs (e.g., see Morgante, Hanafey, & Powell, 2002; Vieira et al., 2016). However, due to widely assumed neutral microsatellite evolution (Freeland et al., 2011; but see also Vieira et al., 2016), independent and local differentiation can lead to genetically distinct microsatellite genotypes between local sites, not showing a general differentiation pattern across sites. This explanation supports our findings of strong genetic differentiation between badlands and grassland sites at location level, but absent differentiation across sites (see Figures 5, 6, 7). Nevertheless, given neutral evolution, one could also expect that sites further apart would be more differentiated than sites within locations (that in some cases are only 1 m apart). In our case, isolation by distance was present though very weak. Therefore, local differentiation caused by strong environmental differentiation between local sites probably counteracts isolation by distance effectively.

Another explanation for the strong genetic differentiation between local sites might be genetic drift (see Freeland et al., 2011). Perchance, few founder *T. praecox* individuals from grassland sites colonized badlands leading to local genetic differentiation patterns. We argue against this explanation because the genetic diversity of badlands individuals was not reduced as it is expected when only one or a few individuals colonize new habitats, leading to a genetic bottleneck event (see, e.g., Nei, Maruyama, & Chakraborty, 1975; Prinz, Weising, & Hensen, 2013). Also, genetic drift involving some dispersed individuals would have led to random differentiation pattern, that is, several differentiated subgroups between local sites, that do not fit the observed relative sharp genetic delimitation between local sites (Figure 7).

## 5 CONCLUSIONS

Our findings indicate small‐scale differentiation in *T. praecox* between badlands and surrounding (semi‐)dry grasslands in the Drei Gleichen region of Thuringia (Central Germany). Despite being only a few meters (1–20 m) apart, we observed significant phenotypic (morphological, functional) differentiation between sites and genetic differences between local sites. Extreme light, temperature, drought, and nutrient stress in badlands compared with grasslands led to environmental heterogeneity among (local) sites, promoting the formation of phenotypically and genetically differentiated *T. praecox* individuals. The close proximity of individuals at both sites prevents stronger differentiation by allowing some genetic exchange. The low number of applied microsatellite markers may limit the strength of the observed genetic differentiation patterns in this study, and significantly more markers should be involved in future work.

In general, recorded phenotypic traits showed morphological and performance‐linked differences, evidently related to environmental differences than explained by location‐specific conditions alone. Common‐garden and transplant experiments should be the next step to test phenotypic trait stability, fitness differences, adaptation through natural selection, and thus ecotype formation.

We demonstrated that desert‐like, dry, and nutrient‐poor badlands and their surrounding (semi‐)dry grasslands are a suitable model habitat for studying phenotypic and genetic differentiation in plants. The worldwide occurrence and the few studies about badlands (e.g., Guàrdia et al., 2000; Regüés et al., 2000, 2017) justify the need for more research on this unique and fascinating habitat type. Our findings contribute to conservation issues addressing the importance of microhabitat conditions for maintaining genetic diversity as the basis for evolutionary processes. Particularly vegetation spreading (expansion of pioneer plants and shrub encroachments) reduces badlands dynamics and thus probably endangering these habitats in the Drei Gleichen region (see, e.g., Moretti & Rodolfi, 2000). Nature conservation authorities are supported by these findings to protect and manage such areas of peculiar microhabitat properties enriching the landscape's diversity.

## CONFLICT OF INTEREST

None declared.

## AUTHOR CONTRIBUTION

K.K. and K.P. together developed the project concept. K.P. obtained approvals from lower nature conservation authorities. K.K. recorded environmental and phenotypic data and took phenotypic and genetic samples during fieldwork. In the laboratory, K.K. analyzed samples and gathered data except stomatal trait data recorded by K.P. Furthermore, K.K. and S.T. statistically analyzed environmental, phenotypic, and genetic data. The manuscript was written with contributions from all authors.

## Data Availability

Phenotypic (morphological, functional), environmental, and genetic raw data will be made available on Dryad data repository (https://doi.org/10.5061/dryad.kwh70rz00). Moreover, we will deposit raw data of functional traits upon publication on the TRY database (http://www.try-db.org).
