# Supplementary material for: Desert‐like badlands and surrounding (semi‐)dry grasslands of Central Germany promote small‐scale phenotypic and genetic differentiation in Thymus praecox
Source: Ecol Evol. 2019 Dec 2;9(24):14066–84. doi: 10.1002/ece3.5844 (PMC6953696; doi:10.1002/ece3.5844)
Supplement: Supplementary file 1 [file ECE3-9-14066-s001.docx]

**Appendix**

**Desert-like badlands and surrounding (semi-)dry grasslands of Central Germany promote small-scale phenotypic and genetic differentiation in *Thymus praecox***

**Kevin Karbstein^1,2*^, Salvatore Tomasello^2^ and Kathleen Prinz^1,3^**

^1^Institute of Ecology and Evolution of Plants, Systematic Botany with Herbarium Haussknecht and Botanical Garden, Friedrich-Schiller-University Jena, Philosophenweg 16, D-07743 Jena, Germany

^2^University of Goettingen, Albrecht-von-Haller Institute for Plant Sciences, Department of Systematics, Biodiversity and Evolution of Plants (with Herbarium), Untere Karspuele 2, D-37073, Goettingen, Germany

^3^Landschaftspflegeverband Suedharz/Kyffhaeuser e.V., Uthleber Straße 24, D-99734 Nordhausen, Germany

**^*^**current address: University of Goettingen, Albrecht-von-Haller Institute for Plant Sciences, Department of Systematics, Biodiversity and Evolution of Plants (with Herbarium), Untere Karspuele 2, D-37073, Goettingen, Germany, kevin.karbstein@uni-goettingen.de

|  | | **Group** | **N** | **Cover Herb Layer** | **Species Richness** | **LAI** | **Slope** | **Slope Exposure** | **Altitude** | **Soil Depth** | **Soil Moisture** | **wT** | **wR** |
| --- | --- | --- | --- | --- | --- | --- | --- | --- | --- | --- | --- | --- | --- |
|  |  |  |  |  |  |  |  |  |  |  |  |  |  |
|  | |  |  | [%] |  |  | [°] | [°] | [m a.s.l.] | [cm] | [%] |  |  |
| **Location** | | Ka | 7 | 47.9 | 13 | 0.6 | **16.3^ab^** | **South^a^** | **292^c^** | **11.0** |  | 5.5 | 7.7 |
|  | | Bu | 6 | 33.5 | 14 | 0.8 | **16.5^b^** | **South^ab^** | **306^bc^** | **10.3** |  | 5.7 | 7.7 |
|  | | Mu | 6 | 34.2 | 13 | 1.3 | **29.2^a^** | **West^c^** | **319^b^** | **27.4** |  | 5.5 | 7.8 |
|  | | Wa | 6 | 37.5 | 11 | 0.9 | **16.0^ab^** | **South-West^bc^** | **350^a^** | **34.0** |  | 5.6 | 7.8 |
|  | |  |  |  |  |  |  |  |  |  |  |  |  |
|  | |  |  | χ²=1.89, p=0.60 | χ²=1.77, p=0.62 | χ²=3.56, p=0.31 | **χ²=10.95, p<0.05** | **χ²=16.21, p<0.01** | **F=31.91, p<0.001** | **χ²=6.66, p=0.08** | - | χ²=1.73, p=0.63 | χ²=2.35, p=0.50 |
|  | |  |  |  |  |  |  |  |  |  |  |  |  |
| **Site** | | B | 12 | **7.2** | **7** | **0.3** | 20.9 | South-West | 323 | **15.2** |  | **5.6** | **7.6** |
|  | | G | 13 | **67.7** | **18** | **1.4** | 17.9 | South-West | 309 | **25.0** |  | **5.4** | **7.8** |
|  | |  |  |  |  |  |  |  |  |  |  |  |  |
|  | |  |  | **t=-12.10, p<0.001** | **W=19, p<0.01** | **t=-7.04, p<0.001** | W=104, p=0.17 | t=0.87, p=0.39 | t=1.44, p=0.16 | **W=32, p<0.05** | - | **W=114.5, p<0.05** | **W=38, p<0.05** |
|  | |  |  |  |  |  |  |  |  |  |  |  |  |
|  | **LME/GLMM** |  |  | **GLMM (Binomial): 37.37; AIC=20.0, p<0.01** | **GLMM (Poisson): 0.93; AIC=164.1, p<0.001** | **LME: 1.73; F_1.20_=58.02, p<0.001** | LME: -0.18; F_1.20_=2.11, p=0.16 | LME: -0.05; F_1.20_=0.95, p=0.34 | **LME: -0.07; F_1.20_=10.62, p<0.01** | **LME: 0.70; F_1.20_=10.01, p<0.01** | - | LME: -0.03; F_1.20_=2.45, p=0.13 | **LME: 0.02; F_1.20_=4.73, p<0.05** |
|  | |  |  |  |  |  |  |  |  |  |  |  |  |
| **Location + Site** | | KaB | 3 | **13.3** | **6** | **0.2** | 20.8 | South | **297** | **8.5** | 15.5 | 5.5 | 7.7 |
|  | | KaG | 4 | **73.8** | **19** | **0.9** | 13.0 | South | **288** | **12.9** | 19.4 | 5.6 | 7.6 |
|  | |  |  |  |  |  |  |  |  |  |  |  |  |
|  | |  |  | **W=0, p<0.05** | **W=0, p<0.05** | **t=-3.68, p<0.05** | W=10, p=0.23 | W=7.5, p=0.69 | **t=3.38, p<0.05** | **t=-2.14, p=0.09** | t=-1.29, p=0.3 | W=-6.5, p=1 | W=10.5, p=0.15 |
|  | |  |  |  |  |  |  |  |  |  |  |  |  |
|  | | BuB | 3 | **5.3** | **8** | **0.3** | 16.9 | South | 308 | **7.5** | 12.6 | 5.9 | 7.6 |
|  | | BuG | 3 | **61.7** | **20** | **1.3** | 16.0 | South | 305 | **13.2** | 13.9 | 5.6 | 7.8 |
|  | |  |  |  |  |  |  |  |  |  |  |  |  |
|  | |  |  | **t=-6.04, p<0.05** | **W=0, p=0.06** | **t=-6.17, p<0.01** | t=0.17, p=0.87 | t=0, p=1 | t=0.23, p=0.83 | **t=-3.84, p<0.05** | t=-0.56, p=0.61 | W=5, p=1 | W=1, p=0.18 |
|  | |  |  |  |  |  |  |  |  |  |  |  |  |
|  | | MuB | 3 | **6.7** | 9 | **0.5** | 27.2 | West | **326** | **13.3** | **29.5** | **5.7** | **7.6** |
|  | | MuG | 3 | **61.7** | 17 | **2.1** | 31.1 | West | **311** | **41.5** | **35.9** | **5.4** | **7.8** |
|  | |  |  |  |  |  |  |  |  |  |  |  |  |
|  | |  |  | **t=-3.73, p=0.06** | t=-1.45, p=0.26 | **t=-6.35, p<0.01** | t=-1.98, p=0.14 | W=8, p=0.18 | **t=2.75, p=0.09** | **t=-3.6, p<0.05** | **t=-6.66, p<0.01** | **W=9, p<0.1** | **W=0, p<0.1** |
|  | |  |  |  |  |  |  |  |  |  |  |  |  |
|  | | WaB | 3 | **3.3** | 6 | **0.3** | **18.6** | South-West | **361** | 31.5 | 19.9 | **5.6** | 7.6 |
|  | | WaG | 3 | **71.7** | 16 | **1.5** | **13.3** | South-West | **339** | 36.4 | 30.5 | **5.2** | 7.9 |
|  | |  |  |  |  |  |  |  |  |  |  |  |  |
|  | |  |  | **t=-5.24, p<0.05** | t=-1.22, p=0.34 | **t=-11.01, p<0.01** | **t=2.76, p=0.05** | t=1.27, p=0.29 | **W=9, p=0.08** | W=4, p=1 | t=-1.95, p=0.18 | **W=2.18, p<0.1** | W=1, p=0.18 |

**Table 1.** Comparison of mean biotic and abiotic environmental factors among locations Kallenberg (Ka), Burg Gleichen (Bu), Mühlburg (Mu) and Wachsenburg (Wa), between badlands (B) and grasslands (G) sites and among local sites (Location + Site) based on 109 *T. praecox* individuals. We calculated medians of Ellenberg indicator values for temperature (wT) and soil reaction (wR). We also performed linear mixed effect models (LMEs) and generalized linear mixed effect models (GLMMs) between environmental factors and sites (location as random factor). The level badlands (B) was taken as a baseline to calculate the effect of grasslands (G) on the particular phenotypic trait. Furthermore, we found marginal significant soil depth differences among locations. However, post-hoc statistics did not detect significant differences between location pairs. Soil moisture is only comparable among local sites (see Material and methods). (Marginal) significant differences are indicated in bold (p < 0.1). N: sample size, wT: Ellenberg indicator value of temperature and wR: Ellenberg indicator value of soil reaction.

|  | **Baseline** | **Group** | **Cover Herb Layer** | **Species Richness** | **LAI** | **Slope** | **Slope Exposure** | **Altitude** | **Soil Depth** | **Soil Moisture** | **wT** | **wR** |
| --- | --- | --- | --- | --- | --- | --- | --- | --- | --- | --- | --- | --- |
|  |  |  |  |  | log() | log() | log() | log() | log() |  | log() | log() |
|  |  |  |  |  |  |  |  |  |  |  |  |  |
| (intercept) |  |  | -0.66, p<0.001 | 1.97, p<0.001 | -1.44, p<0.001 | 2.74, p<0.001 | 5.21, p<0.001 | 5.74, p<0.001 | 1.93, p<0.001 | - | 1.74, p<0.001 | 2.03, p<0.001 |
|  |  |  |  |  |  |  |  |  |  |  |  |  |
| Location |  |  |  |  |  |  |  |  |  |  |  |  |
|  | Bu | Ka | n.s. | n.s. | n.s. | -0.02, n.s. | 0.02, n.s. | **-0.04, p<0.05** | 0.02, n.s. | - | n.s. | n.s. |
|  |  | Mu | n.s. | n.s. | n.s. | **0.63, p<0.01** | **0.35, p<0.001** | **0.04, p<0.05** | **0.79, p<0.05** | - | n.s. | n.s. |
|  |  | Wa | n.s. | n.s. | n.s. | 0.01, n.s. | **0.20, p<0.05** | **0.13, p<0.001** | **0.95, p<0.01** | - | n.s. | n.s. |
|  |  |  |  |  |  |  |  |  |  |  |  |  |
| Site |  |  |  |  |  |  |  |  |  |  |  |  |
|  | B | G | **3.30, p<0.001** | **0.93, p<0.001** | **1.71, p<0.001** | n.s. | n.s. | **-0.04, p<0.01** | **0.71, p<0.01** | - | -0.03, n.s. | **0.03, p=0.09** |
|  |  |  |  |  |  |  |  |  |  |  |  |  |
| Location + Site |  |  |  |  |  |  |  |  |  |  |  |  |
|  |  | BuG | n.s. | n.s. | n.s. | n.s. | n.s. | n.s. | n.s. | - | n.s. | -0.01, n.s. |
|  |  | KaB | n.s. | n.s. | n.s. | n.s. | n.s. | n.s. | n.s. | - | n.s. | 0.02, n.s. |
|  |  | KaG | n.s. | n.s. | n.s. | n.s. | n.s. | n.s. | n.s. | - | n.s. | **-0.04, p=0.06** |
|  |  | MuB | n.s. | n.s. | n.s. | n.s. | n.s. | n.s. | n.s. | - | n.s. | -0.002, n.s. |
|  |  | MuG | n.s. | n.s. | n.s. | n.s. | n.s. | n.s. | n.s. | - | n.s. | 0.003, n.s. |
|  |  | WaB | n.s. | n.s. | n.s. | n.s. | n.s. | n.s. | n.s. | - | n.s. | -0.02, n.s. |
|  |  | WaG | n.s. | n.s. | n.s. | n.s. | n.s. | n.s. | n.s. | - | n.s. | NA |
|  |  |  |  |  |  |  |  |  |  |  |  |  |
| Model Statistics |  |  | **GLM: Binomial. AIC=-27.32** | **GLM: Poisson, AIC = 162.11** | **LM: R^2^=0.64, F_1.23_=41.2, p<0.001** | **LM: R^2^=0.47, F_3.23_=6.18, p<0.01** | **LM: R^2^=0.57, F_3.23_=9.23, p<0.001** | **LM: R^2^=0.88, F_4.20_=37.54, p<0.001** | **LM: R^2^=0.55, F_4.20_=6.14, p<0.01** | - | LM: R^2^=0.10, F_1.23_=2.45, p=0.13 | **LM: R^2^=0.47, F_7.17_=2.11, p=0.10** |

**Table 2.** Results of multiple linear models (LMs) and generalized linear models (GLMs) between environmental factors and locations (Kallenberg (Ka), Burg Gleichen (Bu), Mühlburg (Mu) and Wachsenburg (Wa)), sites (badlands (B) and grasslands (G)) and local sites (Location + Site). In LMs, we log transformed response variables to achieve normality. Results indicate the most important treatment(s) influencing the specific environmental factor. Within modeling procedure, one level of location, sites or local sites (e.g. Bu in location, B in sites, ...) was taken as a baseline to calculate effects of other levels on the explanatory variables (e.g. cover herb layer, species richness). For each level, we provided the slope estimate and the p value (except if the particular treatment (locations, sites, local sites) was removed during modeling procedure). Model statistics specify the performed linear regression model and basic statistics. (Marginal) significant differences are indicated in bold. Non-significant = n.s. = p > 0.1.

**Table 3.** Comparison of mean phenotypic traits among locations Kallenberg (Ka), Burg Gleichen (Bu), Mühlburg (Mu) and Wachsenburg (Wa), between badlands (B) and grasslands (G) sites and among local sites (Location + Site) based on 109 *T. praecox* individuals. We calculated linear mixed effect models (LMEs) and generalized linear mixed effect models (GLMMs) between phenotypic traits and sites (location as random factor). The level badlands (B) was taken as a baseline to calculate the effect of grasslands (G) on the particular phenotypic trait. We found marginal significant patch size differences among locations. However, post-hoc statistics did not detect significant differences between location pairs. (Marginal) significant differences are indicated in bold (p < 0.1). N: sample size, PH: plant height, SLA: specific leaf area, LDMC: leaf dry matter content, SPS: stomatal pore surface and PCI: potential conductance index of abaxial leaf surface.

|  | | **Group** | **N** | **PH** | **Patch Size** | **No. Flowers/Individual** | **Trichome Density** | **Sex (Female Ind.)** | **Phenology (Fruiting Ind.)** | **SLA** | **LDMC** | **SPS** | **PCI** |
| --- | --- | --- | --- | --- | --- | --- | --- | --- | --- | --- | --- | --- | --- |
|  | |  |  | [cm] | [cm²] |  | [%] | [%] | [%] | [mm² mg^-1^] | [mg g^-1^] | [µm²] |  |
| **Location** | | Ka | 40 | **6.1^ab^** | **4507** | **1677^a^** | 42 | **25^a^** | **0^d^** | **13.1^b^** | 328.0 | 407.9 | **3.9^b^** |
|  | | Bu | 36 | **4.9^b^** | **1817** | **1000^b^** | 37 | **3^c^** | **3^c^** | **14.5^a^** | 310.1 | 412.3 | **4.9^a^** |
|  | | Mu | 23 | **6.6^ab^** | **5567** | **762^b^** | 49 | **0^d^** | **33^a^** | **14.1^ab^** | 315.5 | 424.9 | **4.8^a^** |
|  | | Wa | 26 | **7.0^a^** | **4005** | **1043^ab^** | 46 | **21^b^** | **27^b^** | **14.3^ab^** | 317.9 | 401.4 | **3.7^b^** |
|  | |  |  | **χ²=11.3, p<0.05** | **χ²=9.4, p<0.05** | **χ²=14.1, p<0.01** | χ²=5.5, p=0.14 | **χ²=11.0, p<0.05** | **χ²=22.6, p<0.001** | **F=3.3, p<0.05** | χ²=5.5, p=0.14 | F=1.3, p=0.72 | **F=26.6, p<0.001** |
|  | |  |  |  |  |  |  |  |  |  |  |  |  |
| **Site** | | B | 50 | **4.7** | **1551** | 1280 | 43 | 10 | 14 | **13.2** | 322.0 | **401.2** | 4.2 |
|  | | G | 75 | **6.9** | **5349** | 1104 | 42 | 16 | 10.67 | **14.4** | 316.1 | **417.5** | 4.3 |
|  | |  |  |  |  |  |  |  |  |  |  |  |  |
|  | |  |  | **W=953, p<0.001** | **W=851.5, p<0.001** | W=1685.5, p=0.62 | W=1936, p=0.73 | χ²=0.53, p=0.47 | χ²=0.13, p=0.72 | **W=1290, p<0.01** | t=0.87, p=0.39 | **t=-2.69, p<0.01** | t=-0.77, p=0.45 |
|  |  |  |  |  |  |  |  |  |  |  |  |  |  |
|  | **LME/GLMM** |  |  | **LME: 4.95; F_1.120_=24.5, p<0.001** | **LME: 5.84; F_1.119_=34.1, p<0.001** | **GLMM (Poisson): -9.61; AIC=85530.7, p<0.001** | GLMM (Binomial): -0.21; AIC=130.9, p=0.65 | GLMM (Binomial): 0.90; AIC=90.4, p=0.16 | **GLMM (Binomial): -1.70; AIC=90.46, p=0.09** | **LME: 3.14; F_1.120_=9.9, p<0.01** | LME: -5.56; F_1.120_=0.7, p=0.41 | **LME: 0.04; F_1.120_=6.9, p<0.01** | LME: 0.04; F_1.120_=1.8, p=0.18 |
|  | |  |  |  |  |  |  |  |  |  |  |  |  |
| **Location + Site** | | KaB | 20 | 5.3 | **2226** | 1816 | 41 | 20 | 0 | **12.4** | 329.1 | 406.3 | 3.7 |
|  | | KaG | 20 | 7.0 | **6909** | 1529 | 42.5 | 30 | 0 | **13.7** | 326.8 | 409.5 | 4.0 |
|  | |  |  |  |  |  |  |  |  |  |  |  |  |
|  | |  |  | W=154.5, p=0.22 | **W=117, p<0.05** | W=161, p=0.38 | W=192.5, p=0.84 | χ²=0.13, p=0.72 | - | **W=107, p<0.05** | t=0.16, p=0.87 | t=0.28, p=0.78 | t=1.51, p=0.14 |
|  | |  |  |  |  |  |  |  |  |  |  |  |  |
|  | | BuB | 17 | 4.4 | **1098** | 1285 | 39 | 0 | 6 | 13.8 | 314.0 | **399.7** | 4.8 |
|  | | BuG | 19 | 5.3 | **2459** | 744 | 35 | 5 | 0 | 15.1 | 306.7 | **423.6** | 5.0 |
|  | |  |  |  |  |  |  |  |  |  |  |  |  |
|  | |  |  | W=112, p=0.12 | **W = 85, p<0.05** | W=211, p=0.12 | W=185, p=0.37 | χ²=0.0, p=1 | χ²=0.0, p=1 | t=-1.6, p=0.12 | t=0.59, p=0.56 | **t=-2.53, p<0.05** | t=-0.56, p=0.58 |
|  | |  |  |  |  |  |  |  |  |  |  |  |  |
|  | | MuB | 7 | **4.1** | **878** | **343** | 59 | 0 | 40 | 13.9 | 311.7 | 414.2 | 4.5 |
|  | | MuG | 16 | **7.7** | **7619** | **988** | 45 | 0 | 31 | 14.1 | 317.2 | 429.6 | 4.9 |
|  | |  |  |  |  |  |  |  |  |  |  |  |  |
|  | |  |  | **t=-4.0, p<0.001** | **W=3, p<0.001** | **t=-2.16, p<0.05** | W=74, p=0.15 | - | χ²=0.00, p=1 | t=-0.16, p=0.88 | t=-0.36, p=0.73 | t=-1.35, p=0.19 | t=-1.12, p=0.29 |
|  | |  |  |  |  |  |  |  |  |  |  |  |  |
|  | | WaB | 6 | **4.6** | **1367** | **531** | 48 | 0 | **67** | 14.5 | 332.5 | 372.9 | 3.9 |
|  | | WaG | 20 | **7.8** | **4796** | **1197** | 46 | 24 | **15** | 13.5 | 313.5 | 409.9 | 3.7 |
|  | |  |  |  |  |  |  |  |  |  |  |  |  |
|  | |  |  | **t=-4.5, p<0.001** | **W=28, p=0.05** | **t=-2.42, p<0.05** | W=63.5, p=0.84 | χ²=0.00, p=1 | **χ²=3.91, p<0.05** | t=-1.61, p=0.13 | t=1.61, p=0.16 | t=-1.75, p=0.13 | t=1.04, p=0.32 |

|  | **baseline** | **Group** | **PH** | **Patch Size** | **No.Flowers/ Individual** | **Trichome Density** | **Sex (Female Ind.)** | **Phenology (Fruiting Ind.)** | **SLA** | **LDMC** | **SPS** | **PCI** |
| --- | --- | --- | --- | --- | --- | --- | --- | --- | --- | --- | --- | --- |
|  |  |  | log() | log() |  |  |  |  | log() | log() | log() | log() |
|  |  |  |  |  |  |  |  |  |  |  |  |  |
| (intercept) |  |  | 1.44, p<0.001 | 6.82, p<0.001 | 7.16, p<0.001 | n.s. | -4.16, p<0.001 | -3.08, p<0.001 | 2.57, p<0.001 | 310.14, p<0.05 | 5.99, p<0.001 | 1.58, p<0.001 |
|  |  |  |  |  |  |  |  |  |  |  |  |  |
| Location |  |  |  |  |  |  |  |  |  |  |  |  |
|  | Bu | Ka | n.s. | n.s. | n.s. | n.s. | **2.53, p<0.05** | -16.00 | n.s. | 17.85, p<0.05 | n.s. | **-0.24, p<0.001** |
|  |  | Mu | n.s. | n.s. | n.s. | n.s. | -15.13, n.s. | **3.05, p<0.01** | n.s. | 5.40, n.s. | n.s. | -0.02 |
|  |  | Wa | n.s. | n.s. | n.s. | n.s. | 1.67, n.s. | **2.96, p<0.05** | n.s. | 7.76, n.s. | n.s. | **-0.28, p<0.001** |
|  |  |  |  |  |  |  | 0.95, n.s. |  |  |  |  |  |
| Site |  |  |  |  |  |  |  |  |  |  |  |  |
|  | B | G | **0.55, p<0.001** | **1.14, p<0.001** | **-0.07, p<0.001** | n.s. | n.s. | **-1.21, p=0.07** | **0.09, p<0.01** | n.s. | n.s. | n.s. |
|  |  |  |  |  |  |  |  |  |  |  |  |  |
| Location + Site |  |  |  |  |  |  |  |  |  |  |  |  |
|  |  | BuG | **-0.37, p<0.01** | -0.44, n.s. | **-0.47, p<0.001** | n.s. | n.s. | n.s. | n.s. | n.s. | **0.06, p<0.05** | n.s. |
|  |  | KaB | 0.18, n.s. | **0.63, p<0.05** | **0.35, p<0.001** | n.s. | n.s. | n.s. | n.s. | n.s. | 0.02, n.s. | n.s. |
|  |  | KaG | -0.15, n.s. | 0.27, n.s. | **0.25, p<0.001** | n.s. | n.s. | n.s. | n.s. | n.s. | 0.02, n.s. | n.s. |
|  |  | MuB | -0.10, n.s. | -0.31, n.s. | **-1.32, p<0.001** | n.s. | n.s. | n.s. | n.s. | n.s. | 0.04, n.s. | n.s. |
|  |  | MuG | -0.01, n.s. | **0.64, p<0.05** | **-0.19, p<0.001** | n.s. | n.s. | n.s. | n.s. | n.s. | **0.07, p<0.05** | n.s. |
|  |  | WaB | 0.06, n.s. | -0.07, n.s. | **-0.89, p<0.001** | n.s. | n.s. | n.s. | n.s. | n.s. | **-0.07, p=0.06** | n.s. |
|  |  | WaG | NA | NA | NA | n.s. | n.s. | n.s. | n.s. | n.s. | 0.02, n.s. | n.s. |
|  |  |  |  |  |  |  |  |  |  |  |  |  |
| Model Statistics |  |  | **LM: R^2^ = 0.28, F_7.117_=6.5, p<0.001** | **LM: R^2^=0.33, F_7.116_=8.02, p<0.001** | **GLM: Poisson, AIC = 77992** | GLM: Binomial, AIC = 159.09 | **GLM: Binomial, AIC = 84.25** | **GLM: Binomial, AIC = 74.30** | **LM: R^2^=0.08, F_1_._123_=10.69, p<0.01** | LM: R^2^=0.04, F_3.121_=1.63, p=0.19 | **LM: R^2^=0.14, F_7.117_=2.64, p<0.01** | **LM: R^2^=0.38, F_3.121_=25.16, p<0.001** |

**Table 4.** Results of multiple linear model (LMs) and generalized linear model (GLMs) between phenotypic traits of *T. praecox* and locations (Kallenberg (Ka), Burg Gleichen (Bu), Mühlburg (Mu) and Wachsenburg (Wa)), sites (badlands (B) and grasslands (G)) and local sites (Location + Site). In LMs, we log transformed response variables to achieve normality. Results demonstrate the most important treatment(s) (location/site/local sites) influencing the specific phenotypic trait. Within modeling procedure, one level of location, sites or local sites (e.g. Bu in location, B in sites) was taken as a baseline to calculate effects of other levels on the explanatory variables (e.g., plant height, patch size). For each level, we provided the slope estimate and the p value (except if the treatment (locations, sites, local sites) was removed during modeling procedure). Model statistics specify the performed linear regression model and basic statistics. PH: plant height, SLA: specific leaf area, LDMC: leaf dry matter content, SPS: stomatal pore surface, PCI: potential conductance index of abaxial leaf surface. (Marginal) significant differences are indicated in bold. Non-significant = n.s. = p > 0.1.

Table 5. Ln RH test results to check for outlier SSR loci concerning local sites (KaB, KaG, BuB…) based on 109 *T. praecox* individuals. Natural logarithm (ln) ratio of gene diversity [(1 / (1 – H_e(pop1)_))^2^−1] / [(1 / (1−H_e(pop2)_))^2^−1] was calculated for each group combination, and ln RH estimates were standardized to zero mean and standard deviation of one (Schlötterer 2000; Kauer *et al.* 2003). 95 % of the neutral loci are expected between -1.96 and 1.96. Thus, ln RH values outside this range are considered as outliers (Soto-Cerda & Cloutier 2013). Pop1 to 8: 1: BuG, 2: BuB, 3: KaG, 4: KaB, 5: MuG, 6: MuB, 7: WaG and 8: WaB.

|  | **C405** | **D346** | **D347** | **E089** | **E070** | **D257** |
| --- | --- | --- | --- | --- | --- | --- |
| Pop1-Pop2 | -0.22 | 0.55 | -0.26 | -0.63 | -1.02 | 0.04 |
| Pop1-Pop3 | -0.17 | -1.06 | 0.05 | 0.01 | 0.05 | -0.50 |
| Pop1-Pop4 | -0.30 | -0.47 | 0.00 | -0.11 | -0.29 | -0.71 |
| Pop1-Pop5 | -0.77 | -0.55 | 0.69 | -0.36 | -0.37 | 0.40 |
| Pop1-Pop6 | -0.02 | -0.76 | -0.17 | -0.75 | 0.08 | 0.45 |
| Pop1-Pop7 | -1.70 | -0.30 | -0.27 | -0.33 | -0.62 | -0.68 |
| Pop1-Pop8 | -1.12 | -0.21 | 1.42 | 0.11 | -0.28 | 1.00 |
| Pop2-Pop3 | 0.05 | -1.61 | 0.32 | 0.64 | 1.07 | -0.54 |
| Pop2-Pop4 | -0.08 | -1.01 | 0.27 | 0.53 | 0.74 | -0.75 |
| Pop2-Pop5 | -0.55 | -1.10 | 0.95 | 0.28 | 0.65 | 0.36 |
| Pop2-Pop6 | 0.20 | -1.31 | 0.10 | -0.12 | 1.10 | 0.41 |
| Pop2-Pop7 | -1.48 | -0.84 | 0.00 | 0.30 | 0.40 | -0.72 |
| Pop2-Pop8 | -0.90 | -0.76 | 1.69 | 0.75 | 0.75 | 0.96 |
| Pop3-Pop4 | -0.13 | 0.59 | -0.05 | -0.12 | -0.33 | -0.21 |
| Pop3-Pop5 | -0.60 | 0.51 | 0.64 | -0.37 | -0.42 | 0.90 |
| Pop3-Pop6 | 0.15 | 0.30 | -0.22 | -0.76 | 0.03 | 0.95 |
| Pop3-Pop7 | -1.53 | 0.77 | -0.32 | -0.34 | -0.67 | -0.18 |
| Pop3-Pop8 | -0.95 | 0.85 | 1.37 | 0.10 | -0.32 | 1.50 |
| Pop4-Pop5 | -0.47 | -0.09 | 0.69 | -0.25 | -0.09 | 1.11 |
| Pop4-Pop6 | 0.28 | -0.30 | -0.17 | -0.65 | 0.36 | 1.16 |
| Pop4-Pop7 | -1.40 | 0.17 | -0.27 | -0.22 | -0.33 | 0.03 |
| Pop4-Pop8 | -0.82 | 0.26 | 1.42 | 0.22 | 0.01 | 1.71 |
| Pop5-Pop6 | 0.75 | -0.21 | -0.86 | -0.40 | 0.45 | 0.04 |
| Pop5-Pop7 | -0.93 | 0.26 | -0.95 | 0.03 | -0.25 | -1.09 |
| Pop5-Pop8 | -0.35 | 0.34 | 0.73 | 0.47 | 0.10 | 0.60 |
| Pop6-Pop7 | -1.68 | 0.47 | -0.10 | 0.42 | -0.70 | -1.13 |
| Pop6-Pop8 | -1.10 | 0.55 | 1.59 | 0.87 | -0.35 | 0.56 |
| Pop7-Pop8 | 0.58 | 0.09 | 1.69 | 0.44 | 0.34 | 1.69 |

Table 6. Allelic configuration of each sample that is included in the genetic data analysis. Allele sizes are given in base pairs (bp). Locus coverage is the ratio of expected (six loci) to observed number of loci per sample.

| **ID** | **Site** | **Local Site** | **C405** |  |  |  | **D346** |  |  |  | **D347** |  |  |  | **E089** |  |  |  | **E070** |  |  |  | **D257** |  |  |  | **No. of Loci/Sample** | **Locus Coverage** |
| --- | --- | --- | --- | --- | --- | --- | --- | --- | --- | --- | --- | --- | --- | --- | --- | --- | --- | --- | --- | --- | --- | --- | --- | --- | --- | --- | --- | --- |
| KaB_01 | B | KaB | 169 | 173 | 181 |  | 115 | 119 | 121 |  | 130 | 134 | 142 | 162 | 148 | 151 | 166 |  | 153 | 155 |  |  | 102 | 116 | 126 | 130 | 6 | 100.00 |
| KaB_02 | B | KaB | 169 | 173 | 175 |  | 111 | 115 | 121 | 129 | 134 | 140 | 148 | 170 | 148 | 154 |  |  | 153 | 155 |  |  | 100 | 122 | 124 |  | 6 | 100.00 |
| KaB_04 | B | KaB |  |  |  |  | 117 | 119 |  |  | 135 | 140 | 162 |  | 148 |  |  |  | 145 | 149 | 153 | 155 | 94 | 112 | 136 | 140 | 5 | 83.33 |
| KaB_05 | B | KaB |  |  |  |  | 117 | 119 | 123 |  | 130 | 134 | 142 | 170 | 148 | 151 | 157 |  | 149 | 155 |  |  | 104 | 112 | 122 | 134 | 5 | 83.33 |
| KaB_06 | B | KaB |  |  |  |  | 117 | 121 | 127 |  | 140 | 150 | 158 | 170 | 142 | 148 | 151 | 154 | 149 | 152 | 155 | 161 |  |  |  |  | 4 | 66.67 |
| KaB_07 | B | KaB | 169 | 175 | 185 |  | 115 | 119 | 127 | 129 | 130 | 132 | 142 | 144 | 148 | 151 |  |  | 145 | 153 | 155 | 161 | 104 | 114 | 134 | 140 | 6 | 100.00 |
| KaB_08 | B | KaB | 169 | 173 | 175 |  |  |  |  |  |  |  |  |  | 148 | 151 |  |  | 152 | 161 | 165 |  |  |  |  |  | 3 | 50.00 |
| KaB_09 | B | KaB | 173 | 175 | 185 |  | 121 | 125 |  |  | 130 | 135 | 158 |  | 148 | 151 |  |  | 153 | 161 |  |  | 90 | 116 |  |  | 6 | 100.00 |
| KaB_10 | B | KaB | 169 | 175 | 185 |  | 119 | 123 | 125 |  | 132 | 142 | 162 |  | 148 | 151 |  |  | 149 | 155 | 163 |  | 96 | 118 |  |  | 6 | 100.00 |
| KaB_11 | B | KaB | 169 | 173 |  |  | 115 | 119 | 129 |  | 132 | 140 | 144 | 170 | 142 | 148 | 151 | 160 | 145 | 149 | 152 | 161 | 100 | 126 | 134 |  | 6 | 100.00 |
| KaB_12 | B | KaB | 169 | 173 | 175 | 183 | 121 | 123 | 127 |  | 131 | 144 | 170 |  | 148 | 151 | 166 |  | 145 | 153 | 161 |  | 100 | 110 | 120 | 126 | 6 | 100.00 |
| KaB_13 | B | KaB | 169 | 173 | 175 |  | 115 | 121 | 123 | 127 | 136 | 144 | 158 | 170 | 148 | 151 | 154 |  | 141 | 145 | 149 |  | 112 | 114 | 124 | 134 | 6 | 100.00 |
| KaB_14 | B | KaB | 169 | 175 | 179 |  | 115 | 125 | 127 |  |  |  |  |  | 148 | 151 |  |  | 149 | 153 | 159 | 161 | 104 | 114 | 124 | 134 | 5 | 83.33 |
| KaB_15 | B | KaB | 169 | 173 | 177 |  |  |  |  |  |  |  |  |  | 148 | 151 |  |  | 153 | 155 |  |  |  |  |  |  | 3 | 50.00 |
| KaB_16 | B | KaB | 169 | 173 | 175 |  | 115 | 119 | 127 |  | 130 | 134 | 136 | 158 | 148 | 151 |  |  | 149 | 153 | 155 |  | 112 | 114 | 116 | 134 | 6 | 100.00 |
| KaB_17 | B | KaB | 169 | 175 |  |  | 121 | 125 | 127 |  | 130 | 134 | 148 | 160 | 148 | 151 | 160 |  | 153 | 155 |  |  | 100 | 112 | 114 |  | 6 | 100.00 |
| KaB_18 | B | KaB |  |  |  |  |  |  |  |  | 130 | 131 | 136 |  | 148 | 154 |  |  | 149 | 153 | 161 |  | 102 | 112 | 116 | 118 | 4 | 66.67 |
| KaB_19 | B | KaB |  |  |  |  | 115 | 117 |  |  | 130 | 134 | 144 | 170 |  |  |  |  | 149 | 153 | 155 |  | 92 | 116 | 130 |  | 4 | 66.67 |
| KaB_20 | B | KaB |  |  |  |  |  |  |  |  | 134 | 144 | 147 | 170 | 148 | 157 | 160 |  | 145 | 149 | 153 | 155 | 100 | 116 |  |  | 4 | 66.67 |
| KaG_01 | G | KaG |  |  |  |  |  |  |  |  | 136 | 140 | 144 | 170 | 142 | 148 |  |  | 155 | 157 | 163 |  | 100 | 104 | 116 |  | 4 | 66.67 |
| KaG_02 | G | KaG | 169 | 173 | 175 |  | 117 | 135 |  |  | 134 | 150 | 170 |  | 142 | 148 |  |  | 155 | 157 | 163 |  | 100 | 104 | 108 | 118 | 6 | 100.00 |
| KaG_03 | G | KaG | 169 | 173 | 175 | 187 | 113 | 125 | 131 |  | 132 | 142 | 146 | 170 | 148 | 151 | 154 | 160 | 155 | 157 | 163 |  | 90 | 108 | 124 | 126 | 6 | 100.00 |
| KaG_04 | G | KaG | 169 | 171 |  |  | 113 | 115 | 127 | 129 | 131 | 134 | 136 |  | 148 | 160 |  |  | 145 | 155 | 157 | 161 | 106 | 108 | 112 |  | 6 | 100.00 |
| KaG_05 | G | KaG |  |  |  |  | 113 | 119 | 127 | 131 | 136 | 137 | 144 | 148 | 148 | 151 |  |  | 145 | 149 | 157 | 163 | 90 | 96 | 100 | 116 | 5 | 83.33 |
| KaG_06 | G | KaG |  |  |  |  | 117 | 119 | 125 | 129 | 136 | 140 | 170 |  | 148 |  |  |  | 145 | 149 | 155 |  | 116 | 118 | 120 |  | 5 | 83.33 |
| KaG_07 | G | KaG |  |  |  |  |  |  |  |  | 136 | 140 | 170 |  | 148 |  |  |  | 149 | 155 | 157 | 163 | 100 | 112 | 122 |  | 4 | 66.67 |
| KaG_08 | G | KaG |  |  |  |  | 113 | 115 | 119 | 121 | 140 | 170 |  |  | 148 | 151 |  |  | 145 | 149 | 155 | 157 | 100 | 112 | 115 | 136 | 5 | 83.33 |
| KaG_09 | G | KaG |  |  |  |  |  |  |  |  | 130 | 142 | 158 |  | 148 | 151 | 160 |  | 145 | 149 | 155 | 157 |  |  |  |  | 3 | 50.00 |
| KaG_10 | G | KaG | 169 | 173 | 175 |  |  |  |  |  | 130 | 134 | 136 | 146 | 148 | 151 |  |  | 145 | 149 | 155 | 157 | 106 | 112 | 120 | 136 | 5 | 83.33 |
| KaG_11 | G | KaG | 169 | 173 | 175 |  | 113 | 117 | 121 | 129 | 130 | 132 | 140 |  | 142 | 148 | 151 | 154 | 149 | 155 | 161 |  | 100 | 104 | 120 | 142 | 6 | 100.00 |
| KaG_12 | G | KaG |  |  |  |  | 117 | 121 | 131 |  |  |  |  |  | 148 |  |  |  | 155 | 157 |  |  | 106 | 116 | 126 | 142 | 4 | 66.67 |
| KaG_13 | G | KaG | 169 | 181 | 183 |  |  |  |  |  | 132 | 134 | 140 | 142 | 148 | 151 | 160 |  | 149 | 155 | 157 | 163 | 100 | 116 | 126 | 136 | 5 | 83.33 |
| KaG_14 | G | KaG | 169 | 173 | 175 | 181 | 115 | 121 | 123 | 131 | 130 | 135 | 140 | 144 | 148 |  |  |  | 155 | 157 | 163 |  | 100 | 112 | 114 |  | 6 | 100.00 |
| KaG_15 | G | KaG | 169 | 173 |  |  | 121 | 123 | 125 |  | 140 | 144 | 148 |  | 151 | 154 | 157 | 160 | 145 | 155 | 157 |  | 96 | 100 |  |  | 6 | 100.00 |
| KaG_16 | G | KaG | 169 | 173 |  |  | 115 | 117 | 125 | 137 | 142 | 145 | 147 |  | 148 | 154 | 166 |  | 145 | 149 | 155 | 157 | 100 | 114 | 122 |  | 6 | 100.00 |
| KaG_17 | G | KaG |  |  |  |  |  |  |  |  | 128 | 140 | 144 |  | 148 | 160 |  |  | 149 | 155 | 157 | 163 | 100 | 102 | 112 | 116 | 4 | 66.67 |
| KaG_18 | G | KaG | 169 | 173 | 175 |  | 115 | 117 |  |  | 142 | 150 | 170 |  | 148 | 151 | 160 |  | 145 | 149 | 155 | 157 | 96 | 100 | 126 |  | 6 | 100.00 |
| KaG_19 | G | KaG | 169 | 173 |  |  | 115 | 123 | 129 |  | 130 | 134 | 135 | 140 | 148 | 154 |  |  | 155 | 157 |  |  | 102 |  |  |  | 6 | 100.00 |
| KaG_20 | G | KaG | 169 | 173 | 185 | 187 | 113 | 117 | 119 | 123 | 130 | 134 | 140 |  | 148 | 151 | 154 |  | 145 | 149 | 155 |  | 90 | 100 | 108 | 118 | 6 | 100.00 |
| BuB_01 | B | BuB |  |  |  |  | 119 | 121 |  |  | 132 | 135 | 138 | 140 | 148 | 151 |  |  |  |  |  |  |  |  |  |  | 3 | 50.00 |
| BuB_02 | B | BuB | 169 | 173 | 175 |  |  |  |  |  | 129 | 136 | 140 | 142 | 148 | 151 |  |  | 145 | 153 | 155 | 161 | 100 | 116 | 126 |  | 5 | 83.33 |
| BuB_03 | B | BuB |  |  |  |  | 113 | 117 | 127 | 129 | 140 | 142 | 150 |  | 142 | 148 | 154 |  |  |  |  |  |  |  |  |  | 3 | 50.00 |
| BuB_04 | B | BuB |  |  |  |  |  |  |  |  | 122 | 142 | 148 |  | 142 | 148 |  |  | 143 | 149 | 153 | 155 |  |  |  |  | 3 | 50.00 |
| BuB_05 | B | BuB |  |  |  |  | 117 | 121 |  |  | 132 | 135 | 142 | 146 | 142 | 148 |  |  | 153 | 154 |  |  | 96 | 110 | 128 |  | 5 | 83.33 |
| BuB_08 | B | BuB | 173 | 175 |  |  | 117 | 119 | 121 | 125 | 128 | 136 | 142 |  | 142 | 148 | 154 |  | 145 | 149 | 153 | 159 | 100 | 110 | 116 |  | 6 | 100.00 |
| BuB_09 | B | BuB | 169 | 171 | 173 | 175 | 119 | 121 | 127 |  | 136 |  |  |  | 148 | 154 |  |  | 145 | 149 | 153 | 155 | 100 | 114 | 116 | 118 | 6 | 100.00 |
| BuB_10 | B | BuB |  |  |  |  |  |  |  |  | 132 | 134 | 146 |  | 142 | 148 | 160 |  | 155 | 157 |  |  | 94 | 100 | 114 | 124 | 4 | 66.67 |
| BuB_12 | B | BuB | 169 | 173 | 175 |  | 121 | 127 | 133 | 139 | 134 | 140 | 158 | 170 | 148 | 151 | 154 |  | 153 | 155 |  |  | 90 | 100 | 114 | 116 | 6 | 100.00 |
| BuB_14 | B | BuB | 173 | 175 | 183 |  | 117 | 121 |  |  | 136 | 142 | 158 | 166 | 148 | 151 | 154 |  | 153 | 155 |  |  | 100 | 110 | 122 |  | 6 | 100.00 |
| BuB_15 | B | BuB | 169 | 171 | 175 |  | 115 | 117 | 121 |  | 130 | 134 | 144 |  | 142 | 148 |  |  | 145 | 149 | 151 |  | 100 | 110 | 112 | 126 | 6 | 100.00 |
| BuB_16 | B | BuB | 169 | 171 | 175 |  | 115 | 117 | 121 |  | 134 | 142 | 144 |  | 142 | 151 | 157 | 160 | 145 | 155 | 161 | 163 | 116 | 126 |  |  | 6 | 100.00 |
| BuB_18 | B | BuB | 169 | 171 | 175 |  | 115 | 117 | 121 |  | 134 | 135 | 150 | 154 | 142 | 148 | 154 |  | 152 | 153 | 161 |  | 100 | 122 | 130 |  | 6 | 100.00 |
| BuB_19 | B | BuB | 169 | 171 | 175 |  | 115 | 117 |  |  | 130 | 134 | 138 | 142 | 142 | 148 |  |  | 152 | 153 | 159 | 161 | 90 | 112 | 130 | 134 | 6 | 100.00 |
| BuB_20 | B | BuB |  |  |  |  |  |  |  |  | 135 | 136 | 152 | 158 | 142 | 148 | 157 | 160 | 149 | 155 | 157 | 163 | 94 | 116 | 122 | 136 | 4 | 66.67 |
| BuG_01 | G | BuG | 173 | 175 |  |  | 115 | 121 | 127 |  | 130 | 138 | 166 | 170 | 142 | 148 | 151 |  | 149 | 152 | 153 | 161 | 98 | 116 |  |  | 6 | 100.00 |
| BuG_02 | G | BuG | 169 | 175 |  |  | 115 | 117 | 127 | 129 | 134 | 141 | 148 | 150 | 148 | 157 | 160 |  | 153 | 155 | 161 |  | 90 | 100 | 116 | 122 | 6 | 100.00 |
| BuG_04 | G | BuG |  |  |  |  | 115 | 117 | 121 |  | 134 | 140 | 142 | 148 | 142 | 148 | 151 |  | 145 | 152 | 161 | 171 | 90 | 100 | 116 | 132 | 5 | 83.33 |
| BuG_05 | G | BuG | 169 | 173 |  |  | 115 | 119 | 121 | 127 | 122 | 130 | 134 | 142 | 142 | 148 | 151 |  | 145 | 153 | 155 | 161 | 100 | 112 | 118 | 130 | 6 | 100.00 |
| BuG_06 | G | BuG | 169 | 173 | 175 |  | 115 | 117 | 121 | 129 | 134 | 142 | 146 |  | 142 | 148 |  |  | 153 | 161 |  |  | 90 | 114 | 116 |  | 6 | 100.00 |
| BuG_07 | G | BuG | 169 | 173 | 185 |  | 115 | 121 | 129 | 131 | 142 | 144 | 152 | 156 | 142 | 148 |  |  | 145 | 153 | 155 | 161 | 100 | 116 | 118 | 136 | 6 | 100.00 |
| BuG_08 | G | BuG | 169 | 173 |  |  | 113 | 117 | 121 | 127 | 130 | 134 | 142 |  | 148 | 160 |  |  | 145 | 153 | 155 |  |  |  |  |  | 5 | 83.33 |
| BuG_09 | G | BuG | 169 | 175 | 179 |  | 115 |  |  |  |  |  |  |  | 142 | 148 |  |  | 155 | 161 |  |  | 102 | 118 | 122 |  | 5 | 83.33 |
| BuG_10 | G | BuG | 169 | 173 |  |  |  |  |  |  |  |  |  |  | 142 | 148 |  |  | 153 | 161 |  |  |  |  |  |  | 3 | 50.00 |
| BuG_11 | G | BuG | 169 | 173 |  |  | 115 | 117 |  |  | 135 | 141 | 154 | 156 | 148 | 154 |  |  | 157 | 161 | 163 |  | 102 | 104 | 116 |  | 6 | 100.00 |
| BuG_12 | G | BuG | 169 | 173 | 185 |  | 113 | 115 | 121 |  | 134 | 144 | 170 |  | 142 | 148 |  |  | 153 | 161 |  |  | 116 | 128 |  |  | 6 | 100.00 |
| BuG_14 | G | BuG |  |  |  |  | 113 | 121 |  |  | 130 | 134 | 136 |  | 142 | 148 |  |  | 149 | 153 |  |  | 106 | 116 |  |  | 5 | 83.33 |
| BuG_16 | G | BuG | 169 | 175 | 189 |  | 119 | 121 | 123 | 131 | 135 | 148 |  |  | 142 | 148 |  |  | 145 | 153 | 161 |  | 100 | 122 |  |  | 6 | 100.00 |
| BuG_18 | G | BuG | 169 | 173 |  |  | 117 | 121 |  |  | 130 | 135 | 140 |  | 148 | 151 | 154 |  | 149 | 155 | 161 |  | 110 | 134 |  |  | 6 | 100.00 |
| BuG_20 | G | BuG | 173 | 179 |  |  | 113 | 117 | 123 | 129 | 140 | 148 | 158 | 170 | 148 | 151 |  |  | 153 | 161 | 165 |  | 98 | 116 | 134 |  | 6 | 100.00 |
| MuB_01 | B | MuB | 173 | 185 |  |  | 118 | 127 | 129 | 131 | 140 | 144 | 158 |  | 142 | 148 | 151 |  | 153 | 155 | 161 | 163 | 90 | 120 |  |  | 6 | 100.00 |
| MuB_02 | B | MuB | 169 | 173 | 175 |  | 119 | 121 | 131 |  | 135 | 144 | 152 | 160 | 142 | 148 | 151 |  | 153 | 155 | 161 |  | 112 | 116 | 122 | 126 | 6 | 100.00 |
| MuB_03 | B | MuB | 169 | 173 | 175 | 185 | 123 | 131 | 133 |  | 148 | 152 | 160 |  | 142 | 148 | 151 |  | 145 | 153 | 155 |  | 106 | 116 | 122 | 126 | 6 | 100.00 |
| MuB_04 | B | MuB | 169 | 173 | 175 |  | 111 | 117 | 121 | 135 | 128 | 136 | 150 | 154 | 148 | 151 | 154 |  | 153 | 161 |  |  | 112 | 116 |  |  | 6 | 100.00 |
| MuB_05 | B | MuB | 169 | 173 | 175 |  | 117 | 119 |  |  | 136 | 140 | 142 | 170 | 142 | 148 |  |  | 149 | 153 | 155 | 161 | 100 | 106 | 128 |  | 6 | 100.00 |
| MuB_06 | B | MuB | 169 | 173 |  |  | 111 | 117 | 131 | 133 | 144 | 150 | 152 | 154 | 151 | 154 | 160 |  | 153 | 155 | 161 |  | 106 | 112 | 118 |  | 6 | 100.00 |
| MuB_07 | B | MuB | 169 | 173 | 175 | 185 | 113 | 118 | 127 | 131 | 124 | 142 | 160 | 168 | 148 | 154 | 160 |  | 149 | 161 |  |  | 96 | 112 | 116 |  | 6 | 100.00 |
| MuG_01 | G | MuG | 169 | 173 | 175 | 183 | 113 | 118 | 133 |  | 130 | 140 | 152 |  | 142 | 148 |  |  | 149 | 155 | 163 |  | 106 | 116 | 118 |  | 6 | 100.00 |
| MuG_02 | G | MuG |  |  |  |  |  |  |  |  | 130 | 140 | 142 |  | 148 | 154 |  |  |  |  |  |  | 100 | 118 | 124 |  | 3 | 50.00 |
| MuG_03 | G | MuG | 169 | 173 | 175 |  | 111 | 115 | 119 | 133 |  |  |  |  |  |  |  |  | 153 | 155 | 161 |  | 110 | 112 | 118 |  | 4 | 66.67 |
| MuG_07 | G | MuG | 173 | 175 | 183 |  | 113 | 118 | 131 |  | 124 | 133 | 136 |  |  |  |  |  | 149 | 155 |  |  | 90 | 100 | 112 | 122 | 5 | 83.33 |
| MuG_08 | G | MuG | 173 | 175 | 183 |  | 113 | 117 |  |  | 135 | 139 | 142 |  | 148 | 154 | 160 |  | 145 | 151 | 155 |  | 100 | 108 | 116 |  | 6 | 100.00 |
| MuG_09 | G | MuG | 169 | 185 |  |  | 115 | 117 | 121 | 123 | 135 | 141 | 142 |  | 142 | 148 | 151 | 154 | 149 | 153 | 155 |  | 100 | 122 | 136 |  | 6 | 100.00 |
| MuG_10 | G | MuG | 173 | 175 | 181 |  |  |  |  |  | 135 | 141 | 142 |  |  |  |  |  | 145 | 149 | 153 |  |  |  |  |  | 3 | 50.00 |
| MuG_11 | G | MuG |  |  |  |  | 111 | 117 | 129 |  | 130 | 135 | 141 | 142 | 142 | 148 |  |  |  |  |  |  |  |  |  |  | 3 | 50.00 |
| MuG_12 | G | MuG | 173 | 175 | 183 | 185 | 121 |  |  |  | 124 | 137 | 142 |  | 148 | 154 |  |  | 145 | 152 | 163 |  | 116 |  |  |  | 6 | 100.00 |
| MuG_13 | G | MuG | 169 | 175 | 187 |  | 111 | 121 |  |  | 130 | 132 | 140 | 142 | 148 | 154 | 160 |  | 149 | 153 | 161 |  | 90 | 108 | 116 | 128 | 6 | 100.00 |
| MuG_16 | G | MuG |  |  |  |  | 111 | 117 | 121 | 127 |  |  |  |  | 142 | 148 | 160 |  | 149 | 153 | 155 | 161 |  |  |  |  | 3 | 50.00 |
| MuG_20 | G | MuG |  |  |  |  | 117 | 119 | 121 |  | 128 | 130 | 142 | 148 |  |  |  |  | 149 | 155 |  |  |  |  |  |  | 3 | 50.00 |
| WaB_01 | B | WaB | 175 | 183 | 185 |  | 115 | 119 | 121 |  | 139 | 142 | 158 |  | 142 | 148 |  |  | 153 | 157 | 159 |  | 92 | 108 | 116 | 124 | 6 | 100.00 |
| WaB_02 | B | WaB | 177 | 183 |  |  | 115 | 119 | 121 |  | 139 | 142 | 152 | 158 | 142 | 148 | 157 |  | 147 | 153 | 157 |  | 92 | 116 | 124 |  | 6 | 100.00 |
| WaB_03 | B | WaB | 169 | 175 | 181 |  | 117 | 119 |  |  | 124 | 141 | 152 |  | 142 | 148 | 154 |  | 145 | 155 | 157 |  | 96 | 108 | 110 |  | 6 | 100.00 |
| WaB_07 | B | WaB | 169 | 175 | 179 | 181 | 129 | 141 |  |  |  |  |  |  | 142 | 148 | 154 |  | 147 | 153 |  |  |  |  |  |  | 4 | 66.67 |
| WaB_08 | B | WaB | 175 | 179 | 181 |  | 133 | 137 |  |  |  |  |  |  | 142 | 148 | 154 |  |  |  |  |  |  |  |  |  | 3 | 50.00 |
| WaB_10 | B | WaB | 175 | 179 | 185 |  |  |  |  |  |  |  |  |  | 142 | 148 |  |  | 147 | 149 | 153 |  |  |  |  |  | 3 | 50.00 |
| WaG_011 | G | WaG |  |  |  |  | 117 | 121 | 123 |  |  |  |  |  | 148 | 151 | 163 |  | 153 | 155 | 157 |  |  |  |  |  | 3 | 50.00 |
| WaG_02 | G | WaG |  |  |  |  | 115 | 119 | 121 |  |  |  |  |  | 148 | 157 | 160 |  | 153 |  |  |  |  |  |  |  | 3 | 50.00 |
| WaG_03 | G | WaG | 171 | 173 | 185 |  | 111 | 117 | 127 | 129 | 139 | 140 | 142 | 146 | 148 | 151 | 157 |  | 145 | 153 | 155 | 161 | 90 | 92 | 122 |  | 6 | 100.00 |
| WaG_04 | G | WaG | 169 | 173 | 185 |  | 111 | 113 | 115 | 121 | 136 | 152 | 174 |  | 148 | 154 | 160 |  | 145 | 153 | 155 | 161 | 108 | 112 | 116 |  | 6 | 100.00 |
| WaG_07 | G | WaG | 175 | 177 | 183 | 185 | 115 | 121 | 134 | 137 | 130 | 134 | 138 | 140 | 148 | 151 |  |  | 145 | 153 | 157 | 173 | 92 | 100 | 102 | 110 | 6 | 100.00 |
| WaG_08 | G | WaG | 169 | 175 | 185 |  | 115 | 117 |  |  |  |  |  |  | 142 | 148 |  |  | 151 | 153 | 159 | 161 | 102 | 126 |  |  | 5 | 83.33 |
| WaG_09 | G | WaG | 173 | 175 | 185 |  | 117 | 121 |  |  |  |  |  |  | 148 | 151 |  |  |  |  |  |  | 104 | 112 | 122 | 136 | 4 | 66.67 |
| WaG_10 | G | WaG |  |  |  |  |  |  |  |  | 132 | 140 | 150 | 154 | 148 | 151 | 157 |  | 153 | 155 | 161 |  |  |  |  |  | 3 | 50.00 |
| WaG_11 | G | WaG |  |  |  |  | 117 | 121 |  |  |  |  |  |  | 142 | 148 | 151 |  | 149 | 161 |  |  |  |  |  |  | 3 | 50.00 |
| WaG_12 | G | WaG | 169 | 175 | 187 |  | 113 | 117 | 121 | 134 | 130 | 140 | 142 |  | 142 | 148 | 157 |  | 145 | 153 | 161 | 173 | 92 | 94 | 102 | 120 | 6 | 100.00 |
| WaG_14 | G | WaG | 169 | 173 | 187 |  |  |  |  |  | 134 | 144 | 150 | 154 |  |  |  |  | 149 | 155 | 161 |  | 100 | 108 | 116 | 128 | 4 | 66.67 |
| WaG_15 | G | WaG | 173 | 177 | 181 | 183 | 111 | 113 | 115 | 125 | 128 | 132 | 135 |  | 148 |  |  |  | 145 | 155 | 161 | 163 | 100 | 112 | 114 | 118 | 6 | 100.00 |
| WaG_17 | G | WaG | 173 | 181 | 197 |  | 111 | 113 | 115 | 117 | 128 | 134 | 140 |  | 142 | 148 |  |  | 153 | 159 | 163 |  | 104 | 116 |  |  | 6 | 100.00 |
| WaG_18 | G | WaG | 169 | 175 | 183 |  | 111 | 115 | 119 | 121 | 128 | 132 | 156 |  | 148 | 151 | 160 |  | 149 | 153 | 155 | 159 | 92 | 112 | 114 | 122 | 6 | 100.00 |
| WaG_20 | G | WaG | 183 | 187 | 197 |  | 113 | 115 | 117 | 131 | 134 | 138 |  |  | 142 | 148 |  |  | 149 | 153 | 161 | 167 | 106 | 114 | 116 | 128 | 6 | 100.00 |

Table 7. Locus-wise allelic richness (N_A_), mean expected heterozygosity (H_e_) and mean unbiased genetic diversity (uh). Standard error (SE) is given for H_e_ and uh.

|  |  | **C405** | **D346** | **D347** | **E089** | **E070** | **D257** |
| --- | --- | --- | --- | --- | --- | --- | --- |
| **N_A_** | mean | 12 | 18 | 34 | 8 | 18 | 27 |
| **H_e_** | mean | 0.75 | 0.81 | 0.89 | 0.71 | 0.81 | 0.88 |
|  | SE | 0.04 | 0.03 | 0.01 | 0.02 | 0.02 | 0.02 |
| **uh** | mean | 0.20 | 0.21 | 0.13 | 0.24 | 0.16 | 0.16 |
|  | SE | 0.01 | 0.01 | 0.01 | 0.01 | 0.01 | 0.01 |


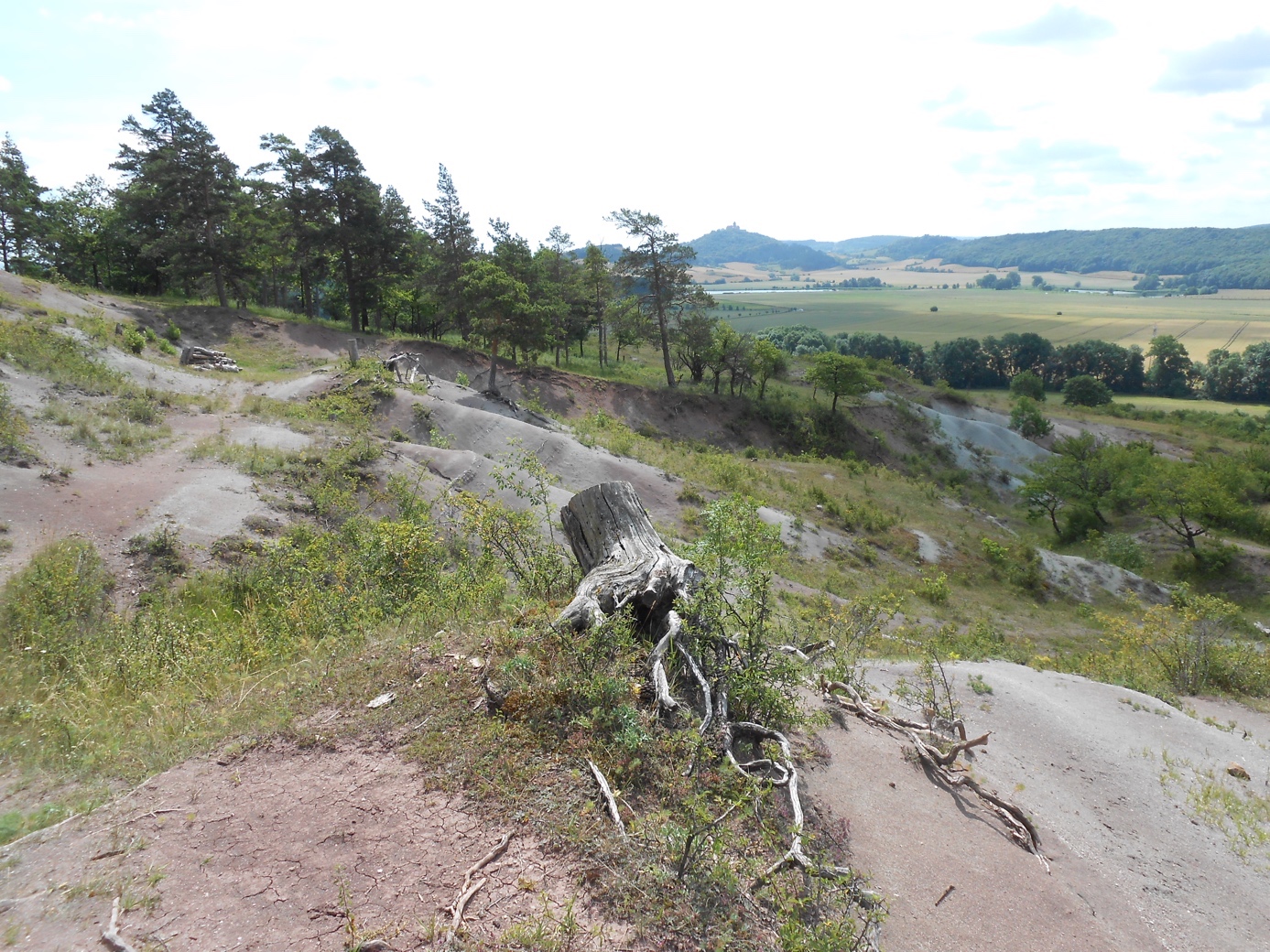


**Fig. 1** Badlands (B) and grasslands (G) at location Burg Gleichen (Bu).


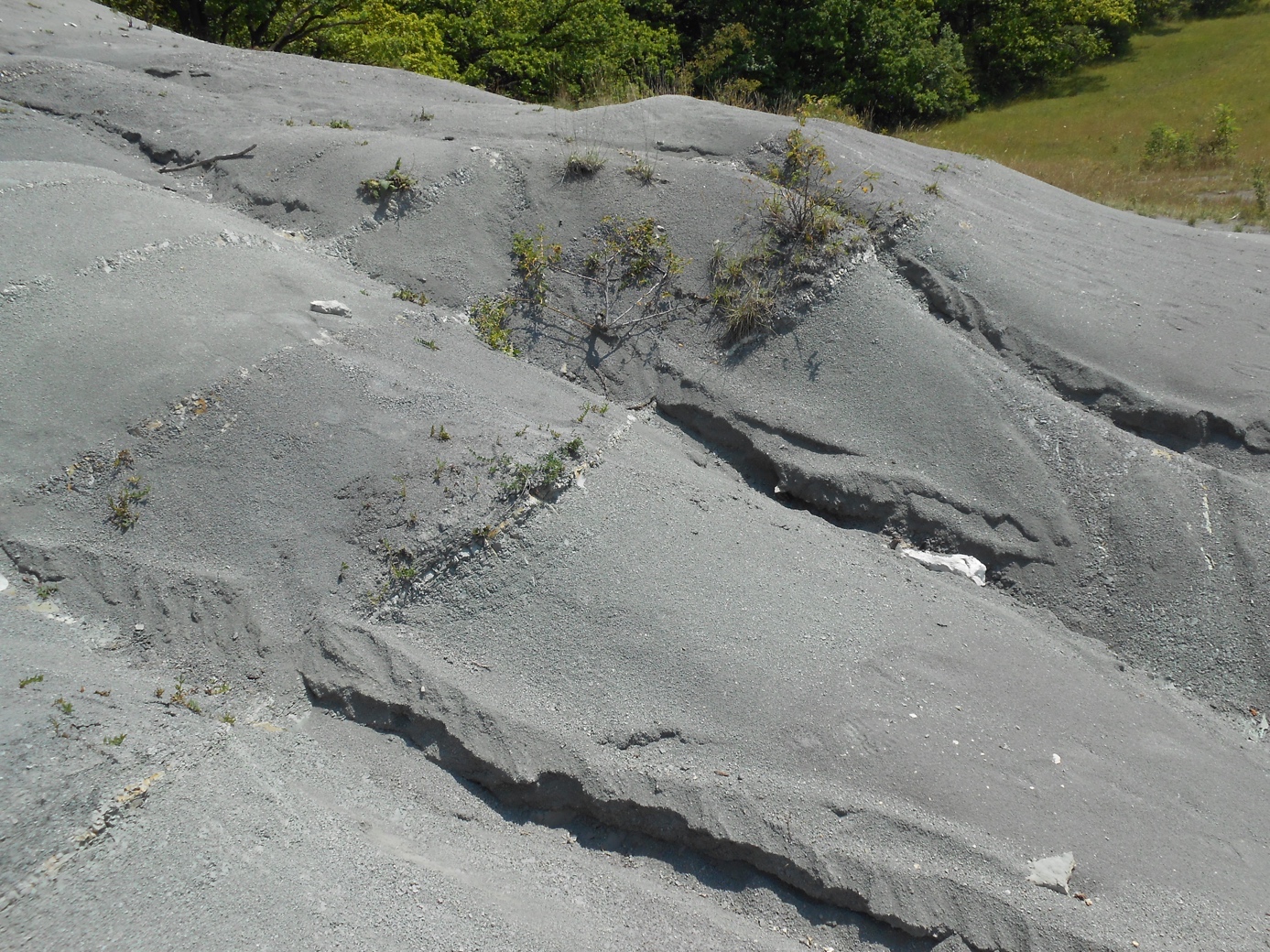


**Fig. 2** Badlands (B) with erosion dunes and gullies at location Wachsenburg (Wa).


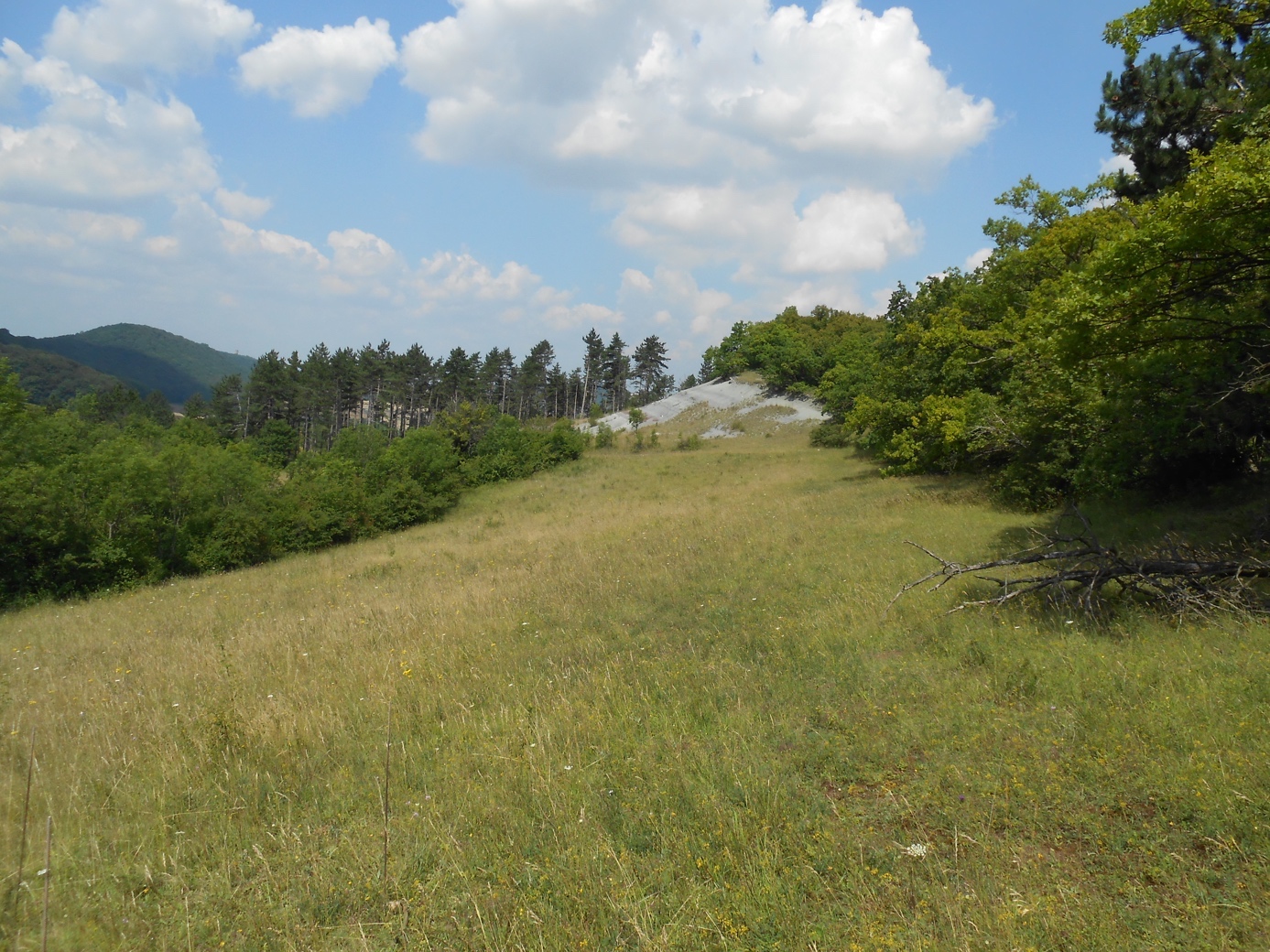


**Fig. 3** Semi-dry and continental dry grasslands (G) surrounding badlands site (B)at location Wachsenburg (Wa).


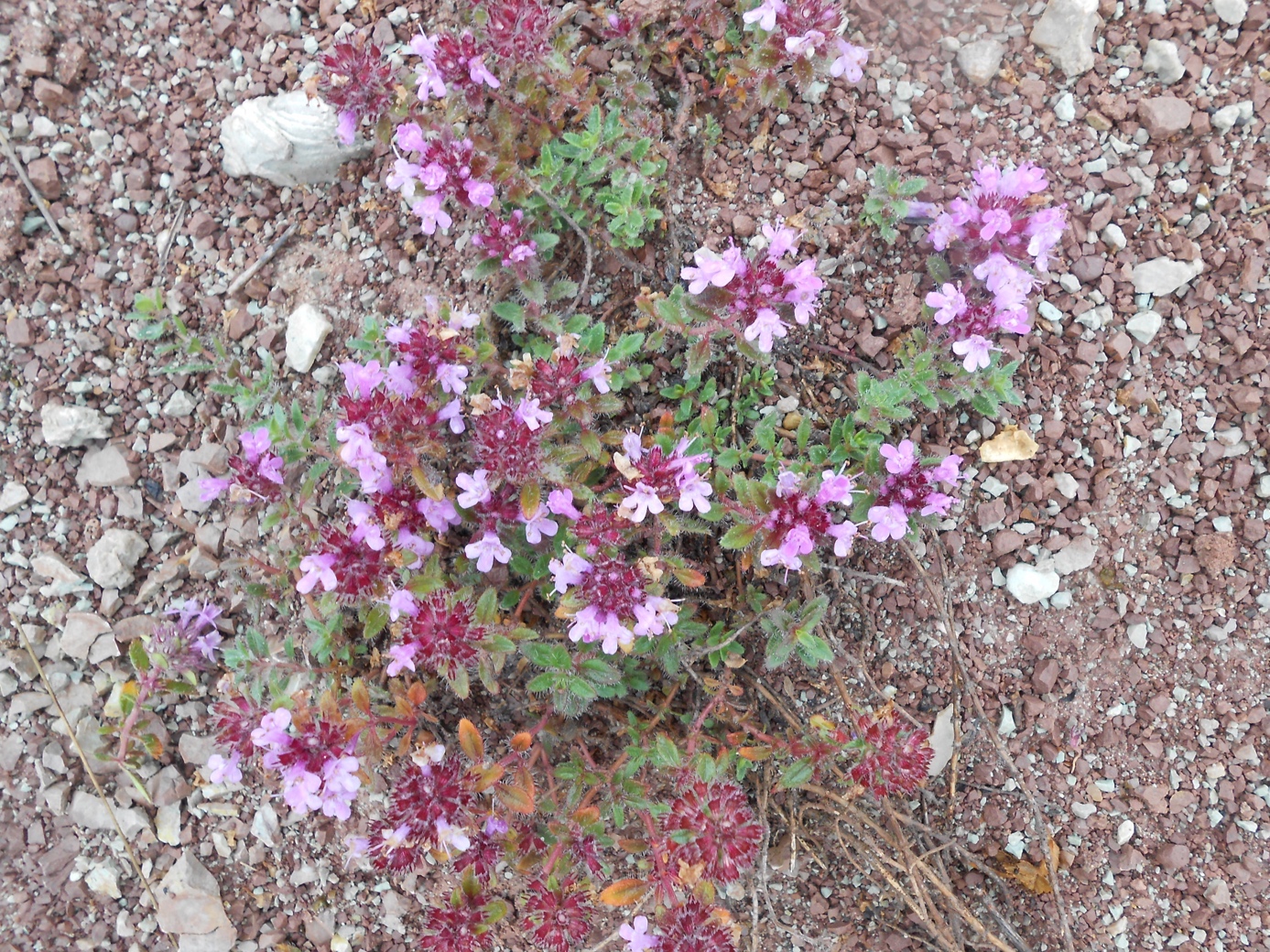


Fig. 4 Hermaphroditic *T. praecox* individual of badlands (B) at location Kallenberg (Ka).

**
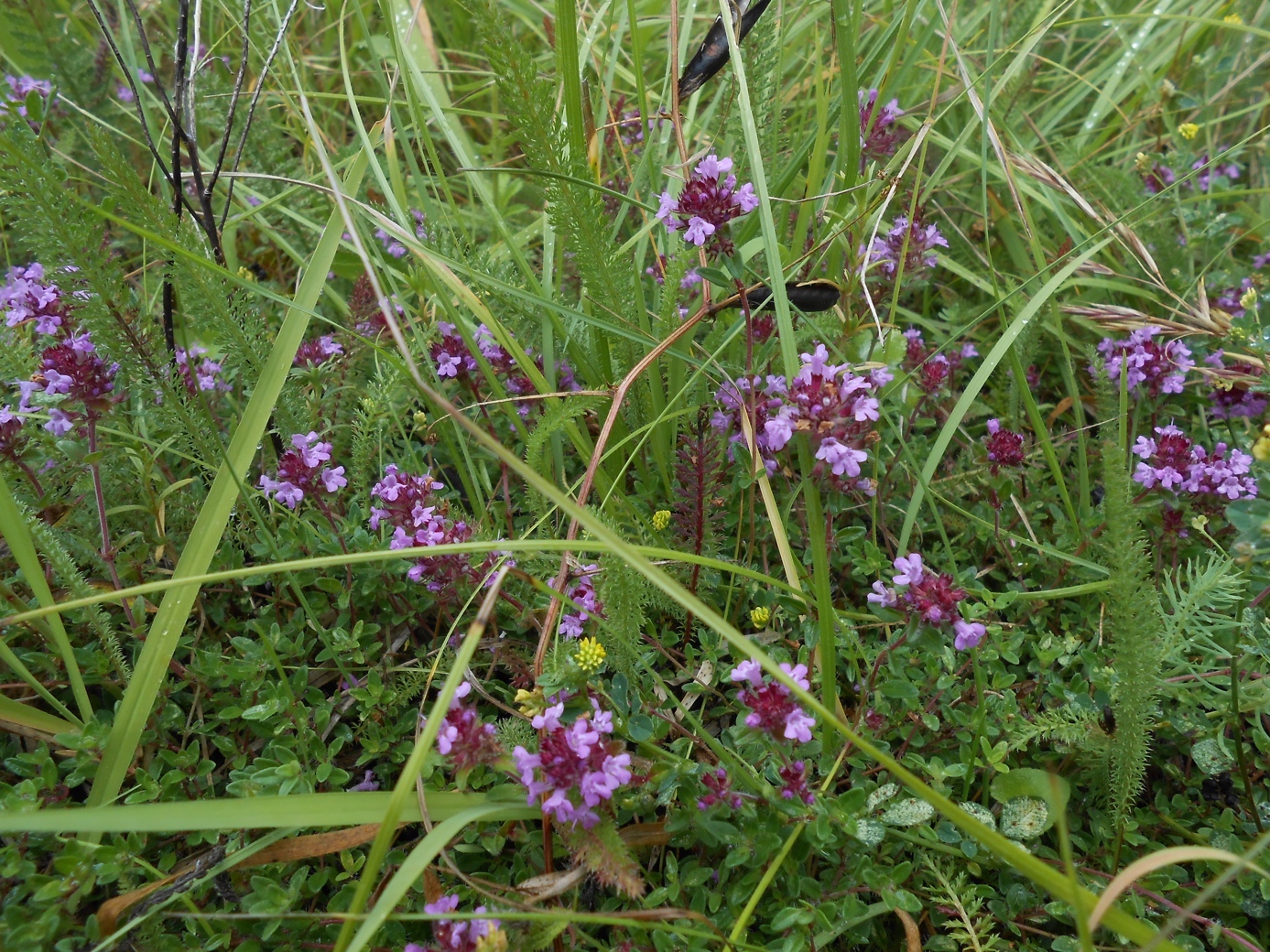
**

Fig. 5 Female *T. praecox* individual of grasslands (G) at location Kallenberg (Ka).


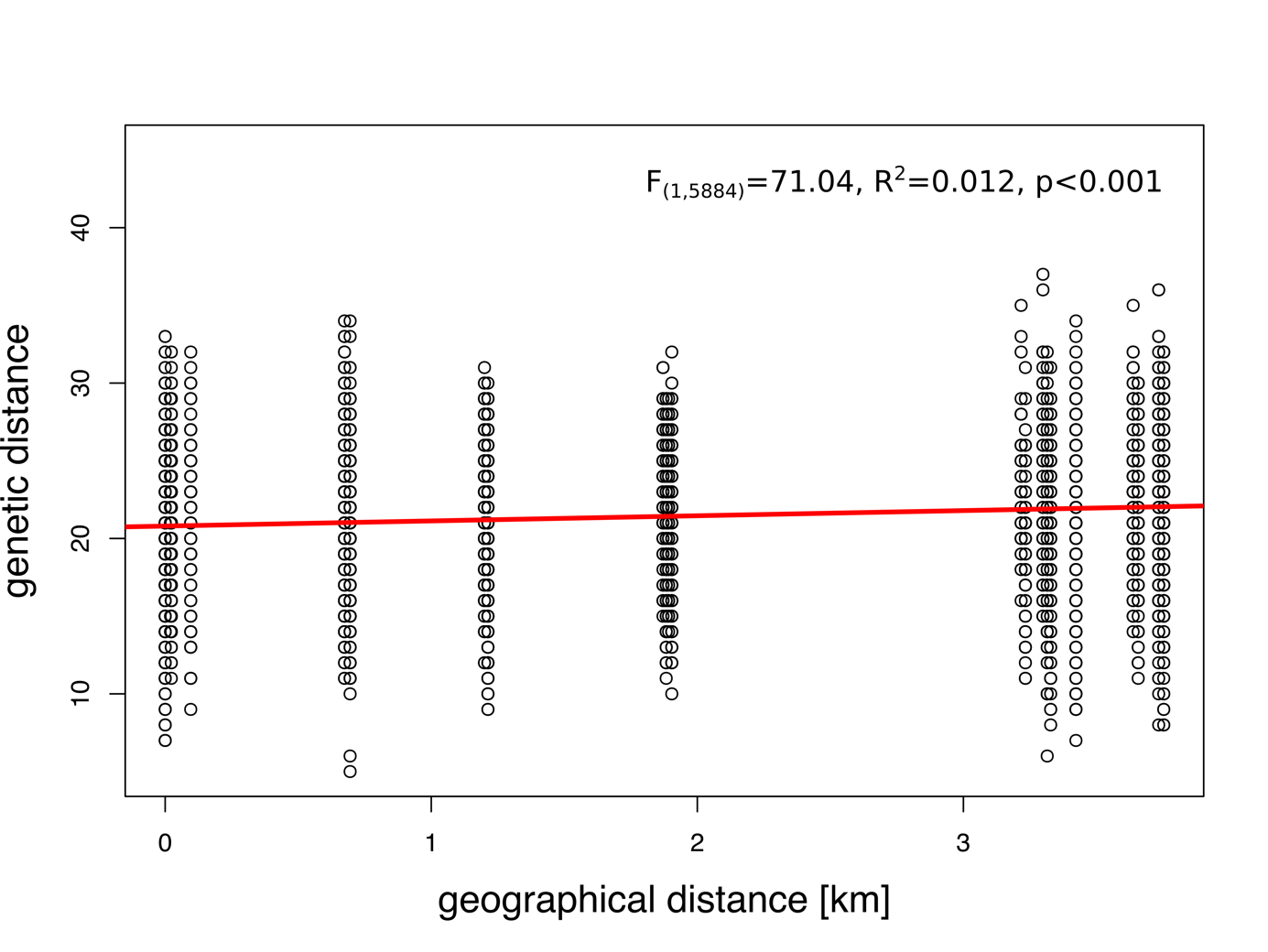
**Fig. 6** Isolation-by-distance (genetic distance in relation to geographical distance) among local sites. We observed a weak but significant isolation-by-distance pattern (R_xy_ *=* 0.109, p = 0.01). We took the geographical coordinates of the center of each local site.

**Table 8.** Pairwise distance matrix (in km) among study locations.

|  | **Kallenberg (Ka)** | **Burg Gleichen (Bu)** | **Mühlburg (Mu)** | **Wachsenburg (Wa)** |
| --- | --- | --- | --- | --- |
| **Kallenberg (Ka)** | - | 0.7 | 1.9 | 4.9 |
| **Burg Gleichen (Bu)** | 0.68 | - | 1.2 | 3.5 |
| **Mühlburg (Mu)** | 1.91 | 1.2 | - | 3.4 |
| **Wachsenburg (Wa)** | 4.85 | 3.5 | 3.4 | - |
